# A comprehensive review on electrospun nanohybrid membranes for wastewater treatment

**DOI:** 10.3762/bjnano.13.10

**Published:** 2022-01-31

**Authors:** Senuri Kumarage, Imalka Munaweera, Nilwala Kottegoda

**Affiliations:** 1 Department of Chemistry, Faculty of Applied Sciences, University of Sri Jayewardenepura, Gangodawila, Nugegoda, Sri Lanka; 2 Instrument Center, Faculty of Applied Sciences, University of Sri Jayewardenepura, Gangodawila, Nugegoda, Sri Lanka; 3 Centre for Advanced Materials Research (CAMR), Faculty of Applied Sciences, University of Sri Jayewardenepura, Gangodawila, Nugegoda, Sri Lanka

**Keywords:** electrospinning, environmental remediation, membrane technologies, nanohybrids, water purification

## Abstract

Electrospinning, being a versatile and straightforward method to produce nanofiber membranes, has shown significant advancement in recent years. On account of the unique properties such as high surface area, high porosity, mechanical strength, and controllable surface morphologies, electrospun nanofiber membranes have been found to have a great potential in many disciplines. Pure electrospun fiber mats modified with different techniques of surface modification and additive incorporation have exhibited enhanced properties compared to traditional membranes and are even better than the as-prepared electrospun membranes. In this review, we have summarized recently developed electrospun nanohybrids fabricated by the incorporation of functional specific nanosized additives to be used in various water remediation membrane techniques. The adsorption, filtration, photocatalytic, and bactericidal capabilities of the hybrid membranes in removing common major water pollutants such as metal ions, dyes, oils, and biological pollutants have been discussed. Finally, an outlook on the future research pathways to fill the gaps existing in water remediation have been suggested.

## Review

### 1 Introduction

Nanotechnology is a technique that exploits the unique properties of matter at dimensions between 1 and 100 nm. Since the discovery of nanotechnology, it has evolved continuously and now has become a technology that is indispensable in diverse disciplines. Among many techniques such as sintering, stretching, track etching, template leaching, and phase inversion [1] to fabricate porous nanomembranes, electrospinning is a straightforward emerging technology that uses electrostatic forces to produce ultrathin fibers with diameters at the nanometer scale. In comparison to the membranes developed via other methodologies, electrospinning provides the manufacturers with membranes of nanometer-scale fibers with high surface area to volume ratio. Also, the low start-up cost, the applicability to a wide range of polymers, the ability to deposit fibers on desired substrates such as metal, glass, microfibrous mats and membranes, and the simple fiber functionalization through blending the functionalizing material with the polymer prior to electrospinning, post-spinning surface functionalization, or the use of a coaxial electrospinning setup make electrospinning a superior technique for membrane fabrication [2].

The smaller size endows the fibers with plenty of exciting properties. The distinctive, exciting properties such as a high surface area to volume ratio, high porosity, interconnected pores, narrow pore size distribution, excellent mechanical, electrical and chemical properties and the tunability of the properties by precise regulation of parameters has made the electrospun nanofibers find its applications in various areas such as the health sector, food, energy and textile industries, and environmental remediation. Electrospun nanohybrids (ENHs) produced by immobilization of function-specific nanoparticles or mixtures of polymers have shown a considerable enhancement of their properties and performances when compared to the as-spun fiber membranes [3–4].

The versatility of electrospinning has allowed for the fabrication of many synthetic and natural polymer membranes [5], and ceramic nanofibers as well [6]. The most common synthetic polymer membranes such as polycaprolactone (PCL), polyacrylonitrile (PAN), polyacrylic acid (PAA), polysulfones (PSF), polyimides (PI), polyvinyl alcohol (PVA), polystyrene (PS), polyethylene oxide (PEO), poly(vinylidene fluoride) (PVDF) and natural polymers such as gelatin, keratin, modified chitin, cellulose acetate (CA) and chitosan (CS) have been electrospun as nanohybrids and have been used in a variety of applications [7–8].

Regarding the health sector, the enhanced biocompatibility, antibacterial properties, and cell compatibility has made ENHs great candidates to deliver bioactive products. Patel et al. fabricated bioactive electrospun nanocomposite scaffolds of poly(lactic acid) for bone tissue engineering by incorporating cellulose nanocrystals and observed that the nanohybrid has excellent properties in terms of mechanical strength and thermal stability compared to the pure electrospun polymer. Also, it has shown superior biocompatibility and osteoinductivity [9]. A nanohybrid electrospun non-woven mat of wool keratin combined with diclofenac loaded hydrotalcites was prepared by Giuri et al. as a bioactive wound dressing [8]. Munaweera et al. developed electrospun CA magnetic fibers by dispersing garnet nanoparticles for magnetically assisted bioseparation [10] and also they developed bandages of ^165^Ho iron garnet nanoparticles incorporated in electrospun PAN to be used against skin cancers [11]. Bugatti and co-workers developed an antimicrobial electrospun hybrid membrane incorporating halloysite nanotubes (HNTs) filled with lysozyme (50 wt % of lysozyme) into Polyamide 11 (PA11) as a bio-based pad for extending the shelf life of chicken slices and has found that the filled nanohybrid membrane resulted in a reduction of bacterial growth compared to electrospun PA11 alone [12]. In addition, ENHs have been utilized in energy applications as well. Zhang et al. developed a graphene oxide (GO)-based nanohybrid Nafion nanofiber as a proton-exchange membrane (PEM) for fuel cells to overcome low proton conductivity, high fuel permeability, and poor stability of predominant PEMs [13]. Zhang et al. developed nanohybrid PVDF membranes by incorporating zeolite with enhanced thermal and electrochemical performance for lithium-ion batteries [14]. ENHs have also been used as a heterogeneous catalyst in indole synthesis by Savva et al. by incorporating gold nanoparticles into cross-linked polyvinylpyrrolidone (PVP) [15].

ENH membranes, along with their high porosity and high aspect ratio, possess a high permeation ability, adsorbability, and selectivity, which makes them excellent for environmental remediation, specifically for the adsorption and filtration of particulate matter. Ge et al. developed an electrospun nanocomposite with rare earth-fused polyurethane to adsorb volatile organic compounds, which are air pollutants [16]. Al-Attabi et al. fabricated a nanohybrid for the submicrometer aerosol particle size filtration by doping wrinkled silica into PAN via electrospinning [17]. Similarly, a wide range of ENH membranes are being used to remove particles, heavy metals, other metal cations, organic chemicals, dyes, and microorganisms from water and will be discussed in detail in this review article.

Water is one of the most critical natural resources and is non-substitutable. Water quality and scarcity are major health and environmental concerns globally. Although 70% of the earth is covered with water, only 2.5% is consumable as fresh water. Despite the scarcity of available fresh water, it is being heavily polluted by industrial effluents, domestic sewage, and agricultural run-offs, leading the world to a critical situation in meeting the growing demand for clean water. When compared with the conventional water treatment methods, ENH membranes have shown promising results to meet these challenges. This article reviews ENH membranes that have been developed in recent years for water purification and treatment. The basics of electrospinning, the limiting factors affecting electrospinning and the advantages of the ENH membranes in water treatment/purification over conventional membranes are highlighted. The application of ENHs in removing major pollutants and their utilization in different membrane technologies for water treatment are then elaborated. Finally, the future potentials of the ENH membranes are discussed.

### 2 Electrospinning technology

Due to its simple operating technique and the superior properties of the produced nanofiber mats, electrospinning has become much more prevalent. The electrospinning apparatus has five components, namely a high voltage supply, a syringe to feed the polymer solution, a metal spinneret or a needle to transfer the polymer solution, a syringe pump to pump the polymer solution from the syringe reservoir and a ground collector to collect the ejected polymer fibers. These are set up inside an enclosure in which ambient parameters, such as relative humidity, airflow, and temperature can be controlled.

A high voltage is supplied to the metal spinneret. The polymer solution, pumped to the spinneret through the syringe, will be charged through this high voltage. Hence, a potential gradient is built up between the polymer droplet at the tip of the spinneret and the ground collector. When the voltage is gradually increased, the hemispherical droplet will transform into a cone, the so-called “Taylor cone”, in which surface tension is dominant. When the voltage is high enough for the electrostatic forces of the Taylor cone to overcome the surface tension and viscous force, jet initiation happens, and a polymer jet will reach the ground collector completing the circuit. Polymers with high molecular weight will form ultrafine fibers due to the long molecular chains. When the molecular weight is low, instead of thin fibers, droplets of the solution will be ejected, which is a phenomenon known as electrospraying [18]. Along its way to the ground collector, the polymer jet undergoes several fluctuations. The jet will initially travel a linear pathway and then will experience bending instabilities with the elongation of the jet due to the electrostatic repulsion forces. This high frequency bending of the polymer jet and the simultaneous evaporation of the solvent produces the ultrathin solid nanofibers collected on the collector [19].

Since the first attempts of electrospinning, the technique has been evolved to become much more efficient and versatile. At present, there are many different facets of electrospinning with slight modifications to the basic setup. These modifications are either based on design modifications, such as multi-jet electrospinning, coaxial electrospinning, emulsion electrospinning, or centrifugal electrospinning, or based on the shape of the collector, such as rotating drum collector, parallel conducting collector, patterned electrodes, rotating thin disk, two-ring collector, and frame collector [20].

### 3 Parameters affecting fiber size, morphology, and structures

The size and the morphology of the electrospun fibers can be controlled by the precise regulation of several parameters, which are classified into three categories, that is, process parameters, solution parameters, and ambient parameters. The process parameters include the applied voltage, the distance from needle tip to collector, and the polymer flow rate. The solution parameters include solution concentration, molecular weight, solution viscosity, volatility of the solvents and solution conductivity. The ambient parameters are the environmental conditions, primarily the relative humidity and the temperature. The effects of these parameters are interconnected, which makes it difficult to separate the impact of each parameter on the fiber properties. And the effect of each parameter can vary for different polymers such that dissimilar results are obtained after changes of parameters.

#### 3.1 Process parameters

**Figure 1:**
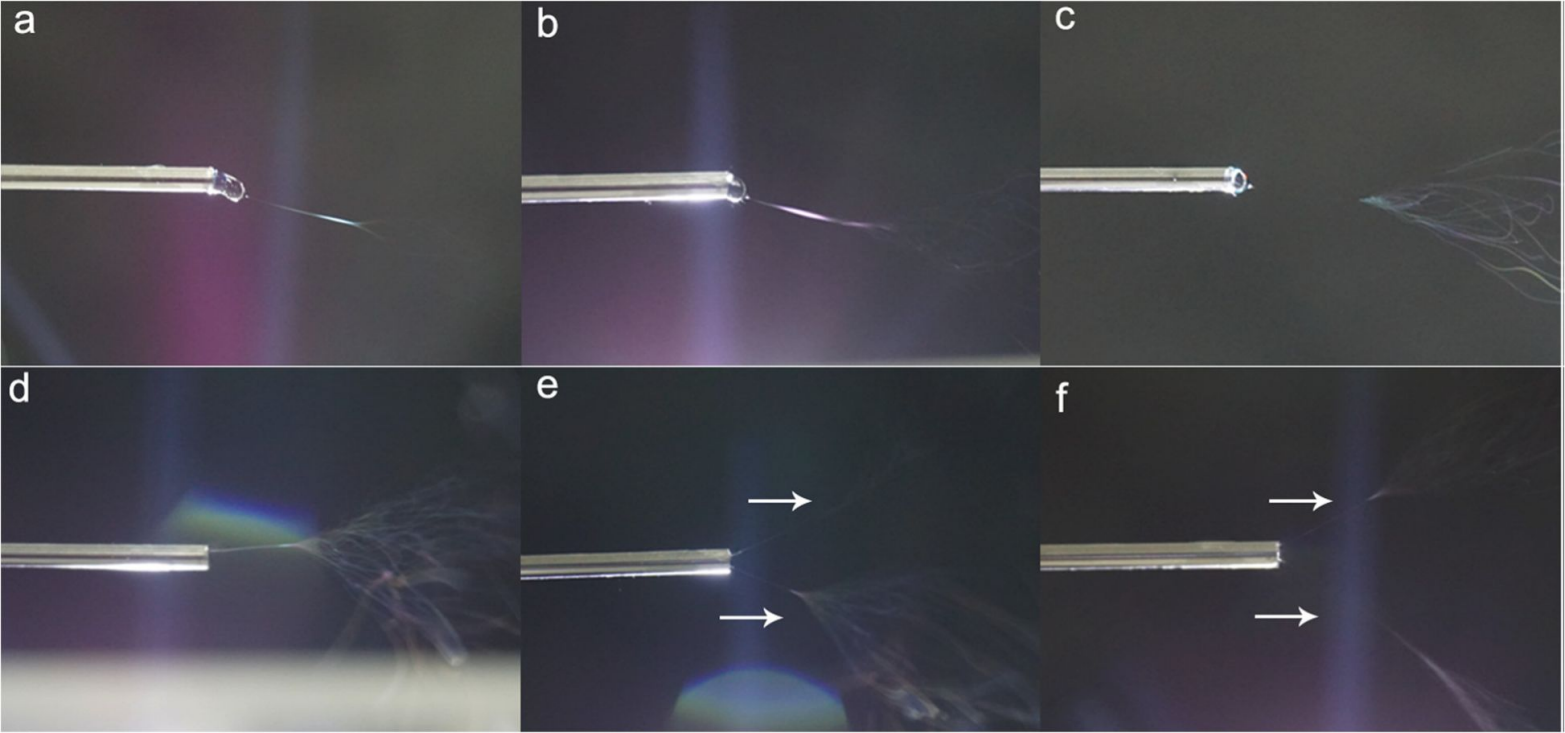
Optical images of jet evolution in spinning process at different voltage values. (a) 6 kV, (b) 10 kV, (c) 15 kV, (d) 20 kV, (e) 25 kV, and (f) 30 kV (the inner diameter of the spinneret is 0.8 mm, the outer diameter is 1.2 mm). Figure 1 was adapted from [23] (© 2019 Z. Liu et al., published by Springer Open, distributed under the terms of the Creative Commons Attribution 4.0 International https://creativecommons.org/licenses/by/4.0/.

**3.1.1 Applied voltage.** The applied voltage is one of the most critical parameters that affect fiber morphology. The applied voltage aids the polymer to overcome its surface tension and form the polymer jet. After forming the Taylor cone, the polymer jet initiation happens when the voltage reaches a threshold value at which the electrostatic forces overcome the surface tension of the polymer droplet. The increase of the applied voltage up to a particular value decreases the fiber diameter [21]. Huan et al. reported that at low voltages (about 10 kV), the electrostatic forces yielded PS/dimethylformamide (DMF) fibers with larger diameters with beaded structures. The authors reasoned that at low voltages the coulombic forces are too low, compared to the surface tension, to elongate the polymer into thin fibers [22]. When a moderate voltage (15 kV) was used, due to the balance of the columbic forces, viscoelastic forces and surface tension, narrow fibers with smooth surfaces were obtained. When a high voltage of 20 kV was used, the fiber diameters were broad and irregular again as the columbic forces were much greater than the viscoelastic forces. Liu and co-workers, using poly(vinylidene fluoride-*co*-hexafluoropropylene) (PVDF-HFP) and PVA, observed that the higher diameter with the increase of voltage is a result of the formation of multiple jets (Figure 1) at the tip of the needle due to a concentrated electrostatic field at the needle tip [23]. The numerous jets will weaken the electrical field per each jet, which hinders the elongation of the polymer into thinner fibers, resulting in thicker fibers and causes spinning instability resulting in wider diameter distribution.

**3.1.2 Nozzle–collector distance.** The distance from nozzle to collector has an effect on the jet flight time and the electric field strength. Similar to the voltage, at first, the increase of distance results in the decrease of fiber diameter, while the further increase of the distance will yield larger fiber diameters. An optimum distance between the nozzle and the collector is essential to provide the time for the solvent to be evaporated and most notably, for the stretching of the fibers due to columbic forces. If the distance is too short, the polymer jet will not have enough time to stretch, which will produce thicker and wet fibers with beads due to the insufficient evaporation of the solvent. If the distance is too high, the weakening of the electrostatic forces hinders the stretching of the polymer yielding thicker fiber diameters [24–25]. Megelski et al. reported bead production in electrospun PS fibers when the nozzle to collector distance was reduced, while the ribbon shaped morphology was preserved [26]. The combination of applied voltage and spinning distance is important. Longer distances allow for a greater time for jet stretching and solvent evaporation at low applied voltages, but they diminish the electric field (*E* = *V*/*D*). The electric field strength, however, is strong at high applied voltages and becomes a dominant factor. The combination of these two factors will define the eventual fiber shape [25].

**3.1.3 Polymer flow rate.** The amount of polymer to be electrospun depends on the polymer flow rate. To obtain a stable Taylor cone, a minimum of polymer solution must be fed to the tip [27]. But when the flow rate is high, an undesirable amount of polymer is fed to the tip of the needle, which increases the fiber diameter. The surplus polymer jet is then difficult to be stretched, and it is difficult for the solvent to be evaporated sufficiently. Also, a pore size increase of a electrospun PVDF membrane incorporated with polydimethylsiloxane has been observed with increased polymer flow rate [28]. Megelski et al. have also reported that the flow rate had an effect on both fiber diameter and morphology for PS in tetrahydrofuran (THF) solvent [26]. They have observed bead formation when the flow rate was 0.10 mL/min and higher. Ribbon-shaped fibers with characteristic microtexture and nanopores increased in size from around 5 to 20 μm. With increasing flow rate, the mean pore size increased from approximately 90 to 150 nm. No effect of the flow rate on the fiber diameters has been observed for CS by Sencadas and co-workers [29].

**Figure 2:**
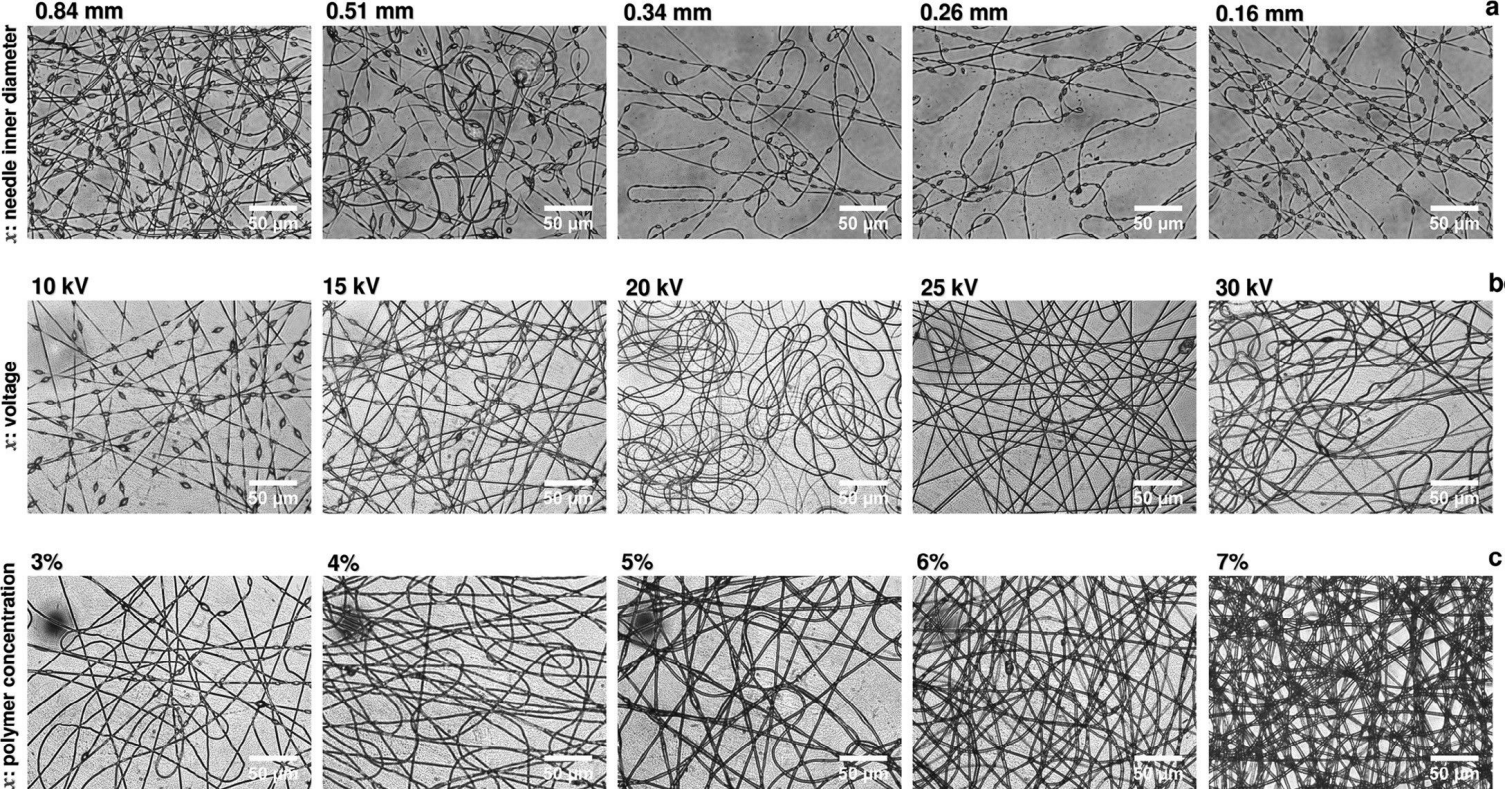
Optical microscopy images of PHB/HAp fibers electrospun under different processing conditions. (a) Polymer concentration of 4%, voltage of 15 kV, inner needle diameter of 0.16–0.84 mm; (b) polymer concentration of 4%, voltage of 10–30 kV, inner needle diameter of 0.34 mm; and (c) polymer concentration of 3–7%, voltage of 25 kV, inner needle diameter of 0.34 mm. Figure 2 was reprinted from [31], Journal of Material Science & Technology, vol. 32, by M. Sadat-Shojai, “Electrospun polyhydroxybutyrate/hydroxyapatite nanohybrids: microstructure and bone cell responser”, pages no 1013–1020., Copyright (2016), with permission from Elsevier. This content is not subject to CC BY 4.0.

**3.1.4 Needle diameter.** The effect of the needle diameter is similar to that of the polymer flow rate. Using a smaller needle diameter, a smaller Taylor cone will be produced at the tip causing thinner fiber jets to be drawn from the needle [30]. Though the fiber diameter decreases with decreasing needle diameter, Sadat-Shojai et al. reported this proportionality might not be valid when the needle diameter is very small [31]. They observed an overall decrease in fiber diameter from 2.3 to 1.8 μm when the needle diameter was decreased from 0.84 to 0.16 mm. But after decreasing the needle diameter from 0.34 to 0.16 mm the fiber diameter range was approximately the same (Figure 2). They have also observed that the needle diameter has no effect on bead formation of the fibers of a polyhydroxybutyrate (PHB)/hydroxyapatite (HAp) composite.

#### 3.2 Solution parameters

**3.2.1 Solution concentration.** It has been observed that there is a direct proportionality between the solution concentration and aspect ratio and fiber diameter. Sadat-Shojai et al. reported that when the polymer concentration of PHB was increased from 3 to 7%, the fiber diameter increased and bead-free smooth fibers were obtained [31]. Huan et al. elaborated these results concerning the viscoelastic properties of the polymer solution [22]. They have observed a similar effect in electrospun PS/DMF with the increase of the PS concentration from 10 to 40%. At low concentrations, they have observed irregular beads of PS on the collector. With the increase of concentration, the beaded structure disappeared and finally, at 40% PS, straight fibers have been observed. The increase of solution concentration increases the viscosity of the solution. At low concentrations due to the low viscosity, the polymer jet cannot withstand the stretching of the solution caused by the electrostatic forces during jet flight, which will lead to bead formation. But when the concentration is increased to an optimum value, the balance of viscoelastic properties yielded smooth fibers [22]. Very high concentrations will yield a highly viscous solution that is impossible to force through the syringe needle. Therefore, it is crucial to maintain an optimum solution concentration for better fiber morphology.

**3.2.2 Solution viscosity.** The solution viscosity, polymer concentration, solvent viscosity and the polymer molecular weight are related linearly. The increase of polymer concentration simultaneously increases the solution viscosity. The lower the viscosity of the solution, the lower the ability of the fluid jet to withstand the electrostatic repulsions, which will produce beads or beaded fibers due to jet fragmentation. These beads on nanofibers progressively shift from spherical to spindle-like forms as the solution viscosity increases resulting in nanofibers with consistent diameters [32]. At the optimum viscosity of the solution, the balance of electrostatic forces, surface tension, and viscoelastic properties leads to bead-free fibers. Further increase of the solution viscosity yields thick fibers with increased diameters while at very high viscosities, the solution is difficult to force through the needle due to the cohesive nature of the highly viscous solutions [29,33].

Fong et al. have reported that after increasing the solution viscosity it is less likely to obtain beads and beaded fibers [34]. They have observed that the diameter of the beads and the average distance between the beads on fibers increases with increasing solution viscosity when electrospinning PEO polymer. Also they have observed gradual shift of the beads from a spherical shape to spindles. Nezarati and co-workers have observed similiar results when electrospinning poly(carbonate urethane); beaded fibers at low viscosities, uniform fibers at an intermediate viscosities and larger fibers at higher viscosities [35]. They hypothesized that at low viscosities the beads form due to inadequate viscoelastic forces to sufficiently suppress the droplet disintegration by the surface tension of the charged jet. In contrast, at higher viscosities, the higher viscoelastic forces prevent the axial stretching during whipping of the polymer jet, resulting in thicker fibers.

**3.2.3 Solvent type.** When choosing a solvent system to prepare the polymer solution, many aspects of the solvent have to be considered. The solvent viscosity, solvent solubility, solvent conductivity, vapor pressure or the volatility of the solvent, and the electrospinnability of the solvents are the foremost parameters to be considered.

Lasprilla-Botero et al. reported that the same polymer yielded different fiber when different solvents were used. This was due to the different physicochemical properties of the solvents, such as the electrical conductivity, inherent viscosity of the polymers, and the difference of solubility parameters of the solvent and the polymer [36]. Moreover, they have reported that entanglement concentrations of the polymers varied significantly with the solvent and a high solubility in the solvents is not the sole factor for obtaining smooth defect-free fibers. Instead, it is both solubility and electrospinnability. They obtained broader fibers with the increase of the solvent viscosity, which directly affects the final solution viscosity, and observed that beaded fibers are obtained when a significant difference exists between the solubility parameters of polymer and solvent. It was ascribed to the weak interactions between the polymer and solvent.

The solvent volatility has a significant effect on porosity and diameter of the fibers. Lin et al. discovered that THF, a solvent with high vapor pressure, formed nanoporous PS fiber sheaths. Decreasing the vapor pressure of the solvent system by adding DMF, a solvent with low vapor pressure, caused the pores to vanish [37]. In contrast, Putti et al. observed porous fibers of PCL when using lowly volatile CHCl_3_ while smooth, nonporous fibers were obtained when using highly volatile THF. The pore formation during electrospinning happens with phase separation, which can occur in two ways, namely thermally induced or vapor-induced, depending on temperature and humidity [38]. Hence, the porous nature of the fibers cannot be explained solely with the solvent volatility, but only in combination with the ambient parameters. Touny et al. observed that the addition of less volatile DMF to highly volatile CHCl_3_ when spinning PLA/HNT nanohybrids causes a decrease of fiber diameter from the microscale to nanoscale from stable fiber jets [39].

**3.2.4 Solution conductivity.** The solution conductivity is one of the major parameters that determine the fiber diameter. In electrospinning, the extension of the fluid jet happens mainly due to the repulsions of the surface charges of the fluid jet. Therefore, the presence of more charges in the fluid jet stretches the polymer into thin nanoscale fibers. Angammana and Jayaram have studied the influence of conductivity on the electrospinning process by adding NaCl salt to the electrospinning PEO/water system [40]. They have detected that the fiber diameter decreases, protrusion-like objects were formed on the fiber surfaces and both the diameter and the number of protrusions enhanced with the increase of conductivity. Furthermore, they have observed multiple jets from a single droplet due to the rise of the local field at the fluid surfaces in the presence of excess ions. Additionally, they have reported that with very high ionic conductivity, the polymer failed to even form the Taylor cone. Uyar and Besenbacher have also observed a similar phenomenon using DMF solvent of different grades having different conductivities [41]. Also, they have shown that even slight changes in the conductivity of the same solvent can affect the fiber structure, resulting in beads or bead-free consistent fibers under same electrospinning conditions. They concluded that at a higher solution conductivity, low concentration PS polymer solutions yield bead-free fibers.

#### 3.3 Ambient parameters

**3.3.1 Relative humidity.** It has been reported that relative humidity has a direct impact on morphology and diameter of the fibers, which in turn affect the mechanical properties of the fibers. Pelipenko et al. investigated the impact of relative humidity on fiber morphology and the mechanical properties of electrospun PVA, PEO, and blends of PVA/hyaluronic acid (HA) and PEO/CS in acetic acid [42]. They have observed that the fiber diameter decreases with increasing relative humidity from 4 to 70%, but with a higher relative standard deviation. Also, at high relative humidity, bead formation was observed. The thinner fibers produced with the increase of relative humidity were stiffer as an effect of the size-dependent surface effect. One of the theories that describe the decrease of fiber diameter with the increase of relative humidity is that the polymer jet dries quickly after exiting the needle under low relative humidity. It is, thus, exposed to voltage-induced stretching only for a brief period of time. At high relative humidities, solidification happens slowly due to the competition between the evaporation of the solvent and the adsorption of water on the hydrophilic polymer. Hence, the longer time of exposure of the polymer jet to voltage-induced stretching will result in thinner fibers [42–43]. In contrast, Huang et al. observed an increase of fiber diameter and decrease in mechanical strength with the increase of relative humidity for PAN and PSF polymers dissolved in DMF [44]. They have also reported that the humidity had an effect on the surface morphology of the fibers. The increase of surface roughness of the fibers due to pore formation has been observed with the increase of relative humidity. The increase of fiber diameter with relative humidity can occur for a variety of reasons. More water molecules are trapped between the needle and the collector when the water partial pressure is higher. Due to molecular polarization, the presence of these water molecules reduces the quantity of surplus charges in the electrospinning jet [45]. As a result, the electric field strength is diminished. In a weaker field, the jets are subject to a lower drawdown force resulting in a smaller elongation.

Ping Lu and Younan Xia have investigated the effect of relative humidity on the porosity of electrospun PS with THF and DMF as solvents [46]. The different volatilities of the solvents, along with the changing relative humidity, yielded both internally and externally porous PS membranes. At low relative humidity (2%), smooth surface morphologies of the fibers were observed, while at a higher humidity (22%, 42%, and 62%), internally and externally porous nanofibers were obtained. DMF, the less volatile solvent, promotes the formation of the internal pores. In contrast, the highly volatile THF promotes the surface pore formation in the presence of water vapor, which does not mix with hydrophobic PS [46].

**3.3.2 Temperature.** The temperature has two opposing effects on the fiber diameters regarding solvent evaporation and solution viscosity. On the one hand, with a decrease in temperature, solvent evaporation slows down, resulting in a longer time for jet solidification. This allows for more time for jet elongation, yielding thinner fibers. On the other hand, the increase of temperature permits further movement of the polymer chains. This results in a lower solution viscosity allowing the fibers to stretch well during spinning, which leads to thinner fibers. It is reported that at low temperatures, the phenomenon of low evaporation is dominant while at high temperatures viscosity decrease predominates. Consequently, at medium temperatures, the fiber diameter goes through a maximum [43]. Pakravan et al. have observed an increase of spinnability of pure CS with an increase of temperature up to a temperature of 80 °C [47]. At room temperature neat CS had very poor electrospinnability and yielded only nanobeads. With the increase of temperature from 40 to 60 °C, the spinnability slightly improved and a combination of beads and fibers was obtained. But at higher temperatures of 80 °C, beads were dominant again. Decrease of viscosity and surface tension may assist in maintaining the spinning jet as temperature rises, while the spinnability improves with quicker drying of the polymer jet and the resultant higher chain entanglement as the solvent evaporates more quickly. At higher temperatures, the rapid drying of the jet, before even being stretched by the electrical field, results in a beaded morphology [47–48].

### 4 Advantage of electrospun nanohybrid membranes for water treatment over conventional membranes

Electrospinning is a newly emerging arena in membrane fabrications. The technique has progressively developed to be used in water purification and treatment. The electrospun membranes have shown potential to overcome the bottlenecks of conventional membranes used in water purification, fabricated by techniques such as phase inversion, sintering, stretching, and track-etching. For instance, sintering and stretching, which are commonly used in fabricating membranes for microfiltration (MF) and membrane distillation (MD) can be applied only to the materials that are chemically stable and can resist high temperatures. Membranes produced by sintering have a low porosity of 10–20% and stretched membranes have weak mechanical properties [19]. Track etching also produces membranes of weak mechanical strength, and the technique is applicable onlyl for a limited number polymers. The track-etched membranes have low porosity and the technique is more expensive than electrospinning [1]. Phase separation is also a versatile membrane fabrication technique. Yet, it has been reported that the interconnected open pore structures and tailorable membrane thickness of electrospun membranes result in superior porosity and permeability compared to nonsolvent-induced phase separation (NIP) and thermally induced phase separation (TIP) membranes [49–50].

Electrospun membranes have shown many advantages over conventional membranes used in water treatment/purification. Some of the common limitations of the conventional membranes are fouling, scaling, limited porosity, low mechanical strength, low permeation, low wettability, and residual solvents in the finally obtained membrane [51]. It is reported that the NIP filtration membranes require more energy to drive the filtration [52]. The use of electrospun membranes consequently decreases the energy consumption by allowing gravity filtration or filtration at lower pressures [53]. Also, the high surface area of the electrospun membranes offers more adsorption sites [54]. Wang et al. developed a highly porous PAN membrane, which was widely tuned by layer-by-layer assembly, which filtered PS microspheres at a lower pressure of 0.6 psi while the pressure required using conventional MF is 10 psi [55]. Su et al. have developed a superhydrophobic PVDF electrospun membrane for MD, which showed exceptional properties [56]. The membrane exhibited a significant antifouling property, stable permeate flux and little scaling or deposition of hard mineral salts on the membrane. In another study Kim and co-workers compared an electrospun PVDF/PMMA MF membrane with a conventionally cast membrane [57]. The electrospun membrane showed higher water permeability and high porosity compared to the cast membrane. Also, the electrospun membrane had a smoother surface, which led to less fouling.

Most of the limitations of the conventional membranes have been overcome by electrospun membranes due to their high porosity, interconnected pores, high surface area, narrow pore size distribution, and due to membrane modification possibilities. The solvent evaporation during spinning also prevents the contamination of the membrane by residual solvents. Hence the electrospun membranes show a tremendous potential in water treatment when compared to the conventional membranes.

### 5 Application of electrospun nanohybrids in water treatment

#### 5.1 Removal of major water pollutants

The major pollutants of the water sources are heavy metals, cations, oils, dyes, and other organic and inorganic chemicals. In recent years, electrospun nanohybrid membranes have been developed and modified for the removal of these pollutants from synthetic/waste water.

Heavy metals and other metal cations, such as Cd^2+^, Pb^2+^, Cu^2+^, Ni^2+^, Hg^2+^, As^3+/5+^, and radioactive metals, such as Th^4+^, U^6+^, are being released to environmental water with the growing industrialization and through agricultural run-offs. Bioaccumulation of these metals causes serious harm to biodiversity and has also become a severe health issue [58]. To remove these heavy metals and other cations, electrospun membranes have been recognized as a promising solution. Most metal ion removing membranes interact with the targeted ions through ionic interactions via functional groups, such as hydroxy groups, carboxyl groups, amino groups, and ester groups, on the membrane surface. Hence, a high surface area and the ability to generate abundant functional group at the surface of the membrane makes electrospun membranes the perfect candidate for metal ion removal.

CS is one of the most commonly used electrospun natural polymers in heavy metal ion removal. Amine functional groups endow CS with metal adsorption capabilities via physisorption. Due to the poor spinnability of CS alone, it is often spun along with another polymer. For instance, a nanofibrous electrospun nonwoven sorbent from CS blended with PEO and phosphorylated nanocellulose (PNC) has been developed by Brandes et al. for the removal of Cd^2+^ from water. The membrane has achieved a maximum adsorption capacity of 232.55 mg/g at 25 °C, which increased with temperature [59]. Surgutskaia et al. also successfully demonstrated the effective removal of Pb^2+^, Cu^2+^ and Ni^2+^ utilizing diethylenetriaminepentaacetic acid (DTPA)-modified CS membranes electrospun in combination with PEO [60]. The maximum adsorption capacities according to the Langmuir model were found to be 177, 142, and 56 mg/g for Cu^2+^, Pb^2+^, and Ni^2+^, respectively. Li and co-workers reported on an electrospun magnetic fluorescence nanofiber membrane from PEO and CS with immobilized carbon quantum dots (CQDs) and Fe_3_O_4_ for the efficient removal of mercury ions from water [61]. The synergistic effect of nanofibrous polymer material and inorganic nanoparticles resulted in a maximum monolayer adsorption capacity of 148 mg/g. The Hg^2+^ sorption reached equilibrium within 100 min. Moreover, the authors developed a simplistic method for real-time and non-invasive tracking of adsorption, based on the linear relationship between adsorption and fluorescence intensity of the membranes. Yari et al. fabricated a novel mesoporous nanohybrid sorbent membrane to remove Pb^2+^ and Cu^2+^ ions from aqueous solutions by electrospinning PVP and 3-mercaptopropyltrimethoxysilane (TMPTMS) infused with cerium oxide [62]. Also, they have modified the PVP/CeO_2_/TMPTMS nanofibers with a surfactant (Pluronic123) to obtain smaller fiber diameters and greater pore volume and average pore diameters. The analysis showed that PVP/CeO_2_/TMPTMS/P123 had a five times larger surface area than PVP/CeO_2_, which resulted in a maximum adsorption capacity of 272.3 mg/g for Pb^2+^ and 263.4 mg/g for Cu^2+^ ion, approximately three times greater than the adsorption capacity of PVP/CeO_2_/TMPTMS alone without surfactant. The sorbent membrane was regenerated by simply using 0.1 M HNO_3_ as a desorbing agent [62]. Electrospun nanohybrids have also been utilized to remove radioactive metals. Talebi et al. investigated the sorption of Th^4+^ and U^6+^ ions by an electrospun nanomembrane of a mixture of PVA, sodium alginate, and PEO with incorporated nano-sized ZSM5 zeolite, which contains hydroxy functional groups (HZSM5). The maximum adsorption values of Th^4+^ and U^6+^ ions were found to be 274.6 and 144.7 mg/g, respectively, at a HZSM5 content of 10 wt %, an adsorbent dosage of 1 g/L, and pH 5.5 [63]. Utilizing the synergistic effect of the oxidation of manganese dioxide and strong adsorption of iron oxides to As^5+^, Aliahmadipoor et al. developed a novel electrospun nanohybrid membrane incorporating inorganic Fe–Mn binary oxide nanoparticles into PVDF for the decontamination of As^5+^. A maximum As^5+^ uptake capacity of around 21.32 mg/g has been attained by the membrane and 70% of the initial adsorption capacity has been regenerated by a diluted alkaline solution [64].

Transportation, food processing, and petrochemical and pharmaceutical industries are the major culprits responsible for oil to spill into water reservoirs. The lipophilic nature of the oil results in the accumulation of oil–water emulsions. Chemical treatments, mechanical recovery, in situ burning, and bioremediation are some of the primary clean-up methodologies for oil spills [65]. ENH membranes have found application as an oil–water separators, owing to the high surface porosity, submicrometer pore sizes, high permeability, and the ability to control the membrane hydrophobicity/hydrophilicity effortlessly. The nanoscale surface roughness of the nanofibers of the membrane has a direct impact on the wetting properties. Oil–water emulsions can be of two types, namely oil-in-water and water-in-oil emulsions, depending on the relative amounts of water and oil. For oil-in-water emulsions, superhydrophilic/superoleophobic membranes are used to permeate water through the membrane while rejecting oil. In the case of water-in-oil emulsions superhydrophobic/superoleophilic membranes are used in which oil is permeated through the membrane while water is rejected [66].

Obaid et al. have electrospun a PSF solution mixed with NaOH nanoparticles in order to obtain a hydrophilic oil separating membrane [67]. The PSF membrane was then modified by a thin surface layer of polyamide (PA), which was obtained from interfacial polymerization of *m*-phenylenediamine (MPD) and 1,3,5-benzenetricarbonyl chloride (TMC). The membrane was used to separate soybean oil from distilled water. The content of NaOH and the thin polyamide layer had a significant effect on the hydrophilicity of the membrane by decreasing the water contact angle from 130° to 13°. A water flux of 5.5 m^3^/m^2^/day, has been achieved by the membrane with the lowest contact angle [67]. Zhang et al. demonstrated the electrospinning of a blend of PLA and poly(3-hydroxybutyrate-*co*-4-hydroxybutyrate) (P34HB), which is a polyester that renders a water-permeable membrane for highly efficient removal of water from the emulsion under gravity filtration. The water permeation time was reduced from 130 to 9 s with the increase of P34HB from 30 to 50 wt % [68]. Ge et al. developed a superhydrophilic and underwater superoleophobic nanofibrous membrane of PAN with hierarchically structured skin constructed by electrospraying silica nanoparticles (SiO_2_ NPs) mixed in a dilute PAN solution on the top surface of an electrospun PAN membrane. The SiO_2_ NPs have been used to increase the nanoscale roughness of the surface, which is a factor that enhances the superwettability. The authors investigated the separation capability of both surfactant-stabilized and surfactant-free oil-in-water emulsions. A permeation flux of 6290 ± 50 LMH and 1120 ± 80 LMH for surfactant-free and surfactant-stabilized emulsions, respectively, was observed and obtained solely under the force of gravity [69].

**Figure 3:**
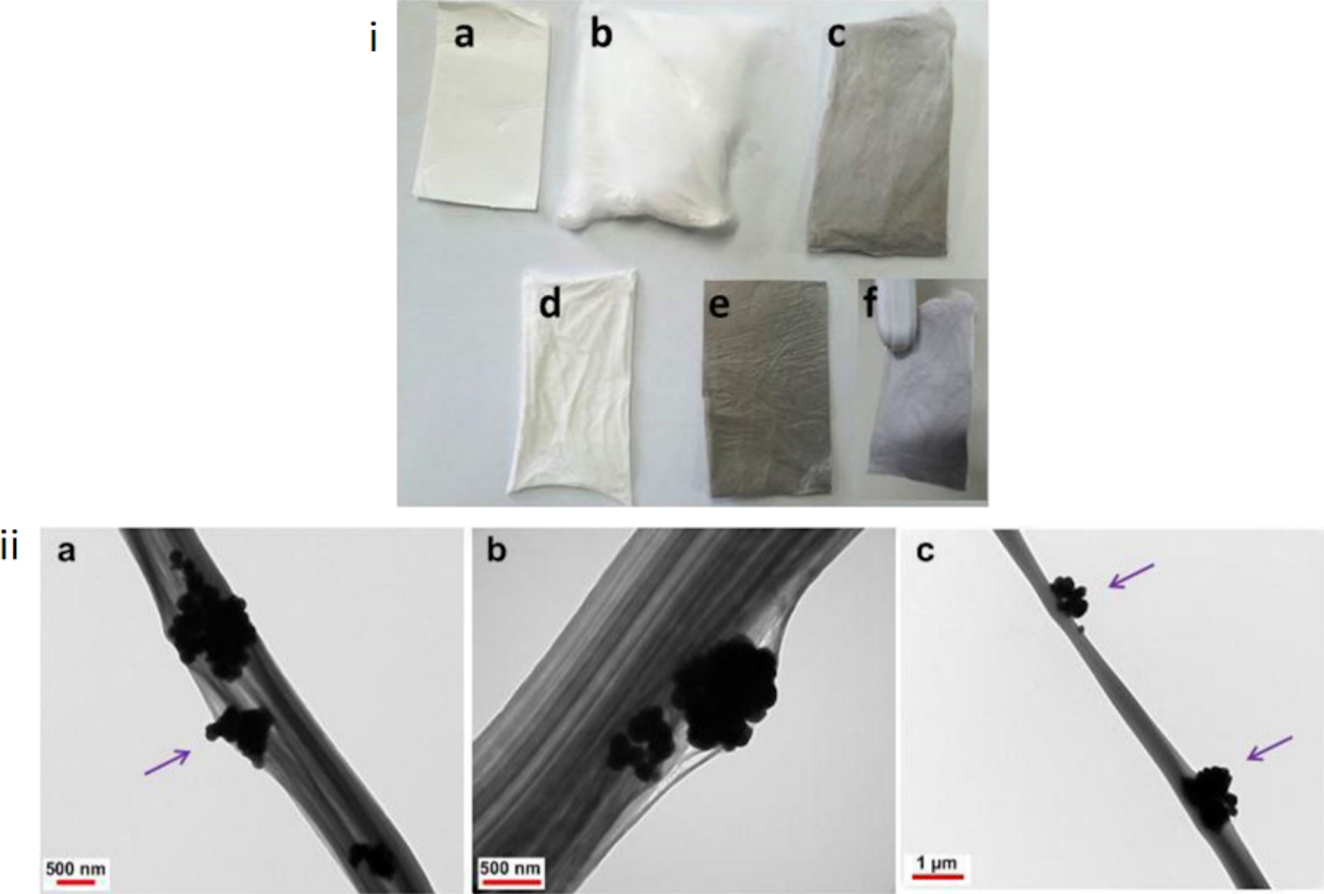
(i) Optical images of the fabricated mats. (a) PVDF, (b) PS, (c) Fe_3_O_4_@PS, (d) PVDF/PS, (e) PVDF/Fe_3_O_4_@PS, and (f) magnetic composite mat when exposed to an external magnet. (ii) TEM images of electrospun Fe_3_O_4_@PS nanofiber at different locations of the nanofiber mat. Figure 3 was reprinted from [72] Composites Part B: Engineering, vol. 77, by Z. Jiang; L. D. Tijing; A. Amarjargal; Ch. H. Park; K.-J. An; H. K. Shon; C. S. Kim, “Removal of oil from water using magnetic bicomponent composite nanofibers fabricated by electrospinning”, pages no 311–318, Copyright (2015), with permission from Elsevier. This content is not subject to CC BY 4.0.

Shang et al. developed superhydrophobic and superoleophilic nanofibrous membranes from electrospun CA with a novel in situ polymerized fluorinated polybenzoxazine (F-PBZ) functional layer with fused SiO_2_ NPs. The prepared nanohybrid showed efficient separation of oil and water with excellent stability in the range of pH 2–12, indicating its potential to be used in oil spill cleanup and the treatment of industrial oil-polluted water [70]. Ma and co-workers also fabricated an oil–water separating nanohybrid membrane with a SiO_2_ NP-integrated F-PBZ functional layer on the surface of an electrospun core–shell-structured membrane of CA/PI nanofibers. The membrane showed hydrophobicity with a water contact angle of 160° and superlipophilicity with an extremely low oil contact angle of 0°. The gravity-driven oil separation through the membrane was fast with a separation efficiency greater than 99% [71]. Jiang et al. fabricated a PVDF/PS magnetic nanofibrous membrane by selective inclusion of Fe_3_O_4_ nanoparticles in PS via a two-nozzle electrospinning process (Figure 3). Integration of magnetic Fe_3_O_4_ NPs to the composite mat helped in the easy recovery of the mats after application in oil–water separation, while PVDF provided mechanical strength. The membrane exhibited an oil sorption capacity of 35–46 g/g for four types of oils, namely sunflower oil, soybean oil, motor oil, and diesel oil [72]. Another superhydrophobic, superoleophilic oil–water-separating electrospun membrane has been successfully fabricated by Reshmi et al. with beeswax and PCL. They have evaluated the separation of a variety of oil–water mixtures such as petrol–water, diesel–water, kerosene–water, gingelly oil–water, and sunflower oil–water. The beeswax/PCL hybrid nanomembrane was then analyzed regarding sorption capacity and separation efficiency in gravity filtration. It showed higher sorption capacity for gingelly oil (25.17 g/g) and sunflower oil (31.05 g/g) than for petrol (19.38 g/g), kerosene (20.72 g/g) and diesel (16.95 g/g). Moreover, the membrane exhibited a high flux and high separation efficiency of 98.1% [73].

Organic dyes are complex structures with high molecular weight, predominantly with ring structures, and can be anionic or cationic. Direct red 23 (DR23), indigo carmine (IC), congo red (CR), and eriochrome black T (EBT) are anionic dyes, while basic blue 41 (BB41), toluidine blue O (TBO), methylene blue (MB), methyl violet (MV), and rhodamine blue (RhB) are cationic organic dyes [74]. Textile, tannery, paper, cosmetics, pharmaceuticals, and food and paper industries are responsible for the release of dye-containing effluents into the aquatic environments. It is reported that 200 billion liters of dye-containing effluents are produced annually by the textile industry alone, and approximately 50% of the effluent is cleared directly into the waterways [75]. Though organic dyes have been considered as a micropollutant, with the growing industrialization, organic dyes have become a significant issue in wastewater treatment. Whether the dye is cationic or anionic, electrospun nanohybrid membranes are used to treat a variety of dye-contaminated waters.

Of the many methods of dye decontamination of water, membrane adsorption has been found to be the most efficient. For example, a nanohybrid membrane of PVA/CS crosslinked with glutaraldehyde vapor and incorporated with SiO_2_ has been prepared by Hosseini and co-workers [76]. They investigated the removal of organic dyes and found that the incorporation of 1.0 wt % SiO_2_ rendered optimum water permeability and dye rejection. A 98% dye rejection of DR23 was shown by the optimum nanofiber composite when operated for 20 min under 1711 LMH (under 0.4 bar applied pressure) of high water flux [76]. The same group fabricated a clay-based electrospun nanohybrid membrane by incorporating montmorillonite (Mt) into CS and PVA mixed nanofibers to be used as affinity membranes for dye removal with ultrafast permeating adsorption. With 2 wt % of Mt, a high water flux of 1765 LMH under 0.4 bar operating pressure and a dye removal efficiency of 95% of BB 41 was achieved. The membrane also showed antifouling properties and was reusable for several dye removing cycles [77]. Furthermore, Gopi and co-workers reported a PVDF electrospun nanomembrane functionalized with chitin nanowhiskers (ChNW) using a needleless electrospinning technique. ChNW transforms the hydrophobic PVDF membrane to a hydrophilic membrane with a water contact angle of 22.72°. The nanohybrid membrane showed a dye removal efficiency of 88.9% and an adsorption capacity of 72.6 mg/g for IC [78]. Huong et al. developed a waste protein-immobilized cationic dye removal membrane from PAN. In this work, a mildly hydrolyzed PAN membrane has been modified by fusing bovine serum albumin (BSA) obtained from laboratory wastes, and it has shown a maximum dye removal capacity of 434.78 mg/g, at pH 12 for TBO. The membrane has the ability to be regenerated by eluting the dye completely with 1 M NaCl or 50% glycerol. 97% of the dye removal efficiency of the membrane was maintained even after five consecutive adsorption/desorption cycles [79]. Hou et al. added the photoactivity of TiO_2_ into a hybrid membrane of PVA, PAA, and carboxyl-functionalized GO to degrade organic dyes by photocatalytic degradation. The membrane displayed an efficient photocatalytic capacity for MB, CR, and RhB [80]. Although TiO_2_ is abundant and inexpensive, it only converts to UV part of sunlight, which is only 5% of the solar energy. This makes the use of TiO_2_ impractical. To counter this drawback Liu and co-workers incorporated Ag NPs into the same composite of PVA, PAA, and carboxyl-functionalized GO. The resulting membrane showed a remarkable photocatalytic decomposition efficiency for MB even after eight catalytic cycles at room temperature [81].

**Figure 4:**
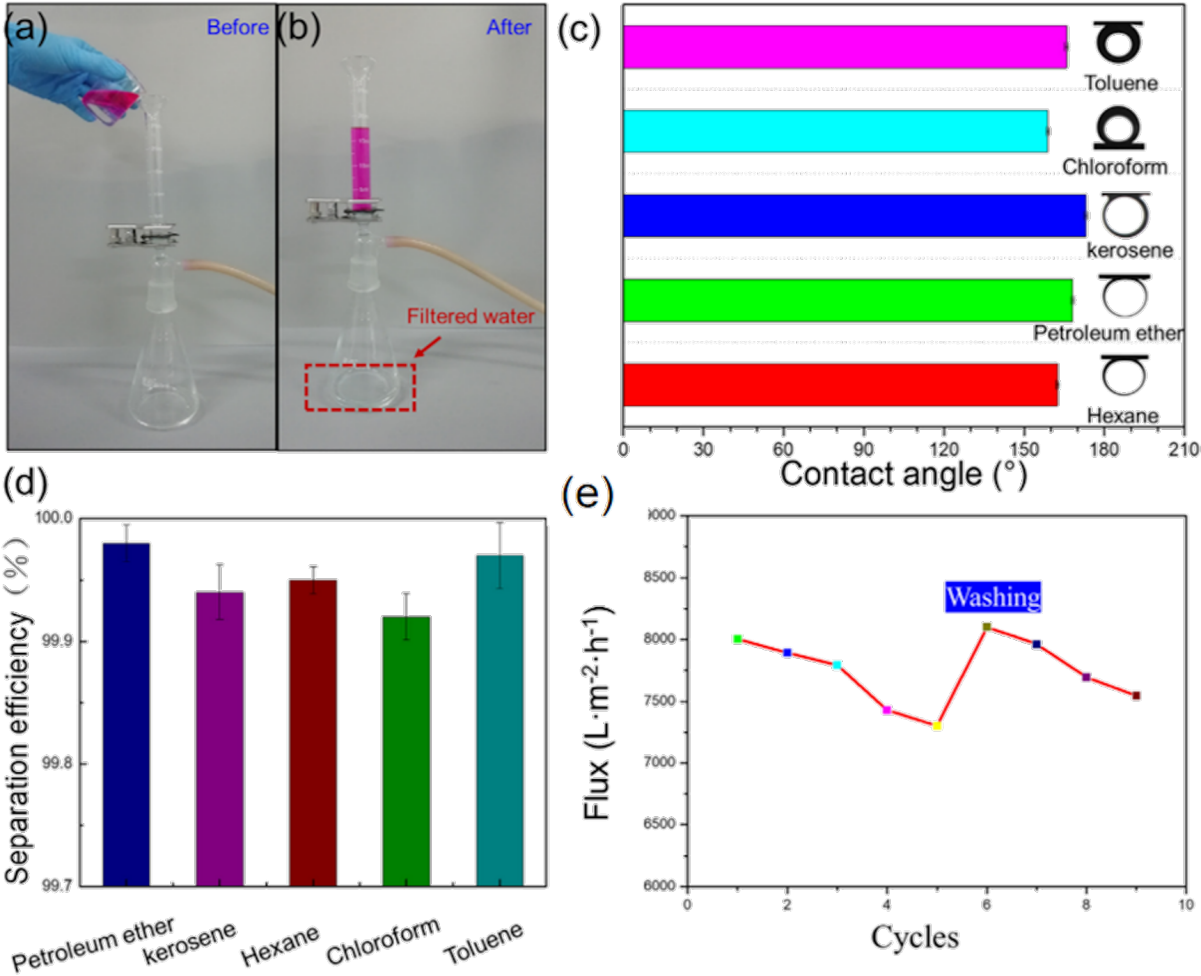
Highly efficient removal of organic chemicals or oils from water (the organic chemicals or oils dyed with red color) (a, b), underwater contact angles of various oils and organic chemicals on the surface of a Pd-decorated polydopamine-SiO_2_/PVA nanofiber membrane (c), separation efficiencies for various organic chemicals or oil–water mixtures (d). Flux for hexane using a Pd-decorated polydopamine-SiO_2_/PVA nanofiber membrane (e). Figure 4 was reprinted from [83], Journal of Cleaner Production, vol. 275, by H. Yin; J. Zhao; Y. Li; L. Huang; H. Zhang; L. Chen, “A novel Pd decorated polydopamine-SiO_2_/PVA electrospun nanofiber membrane for highly efficient degradation of organic dyes and removal of organic chemicals and oils”, article no. 122937, Copyright (2020), with permission from Elsevier. This content is not subject to CC BY 4.0.

**Figure 5:**
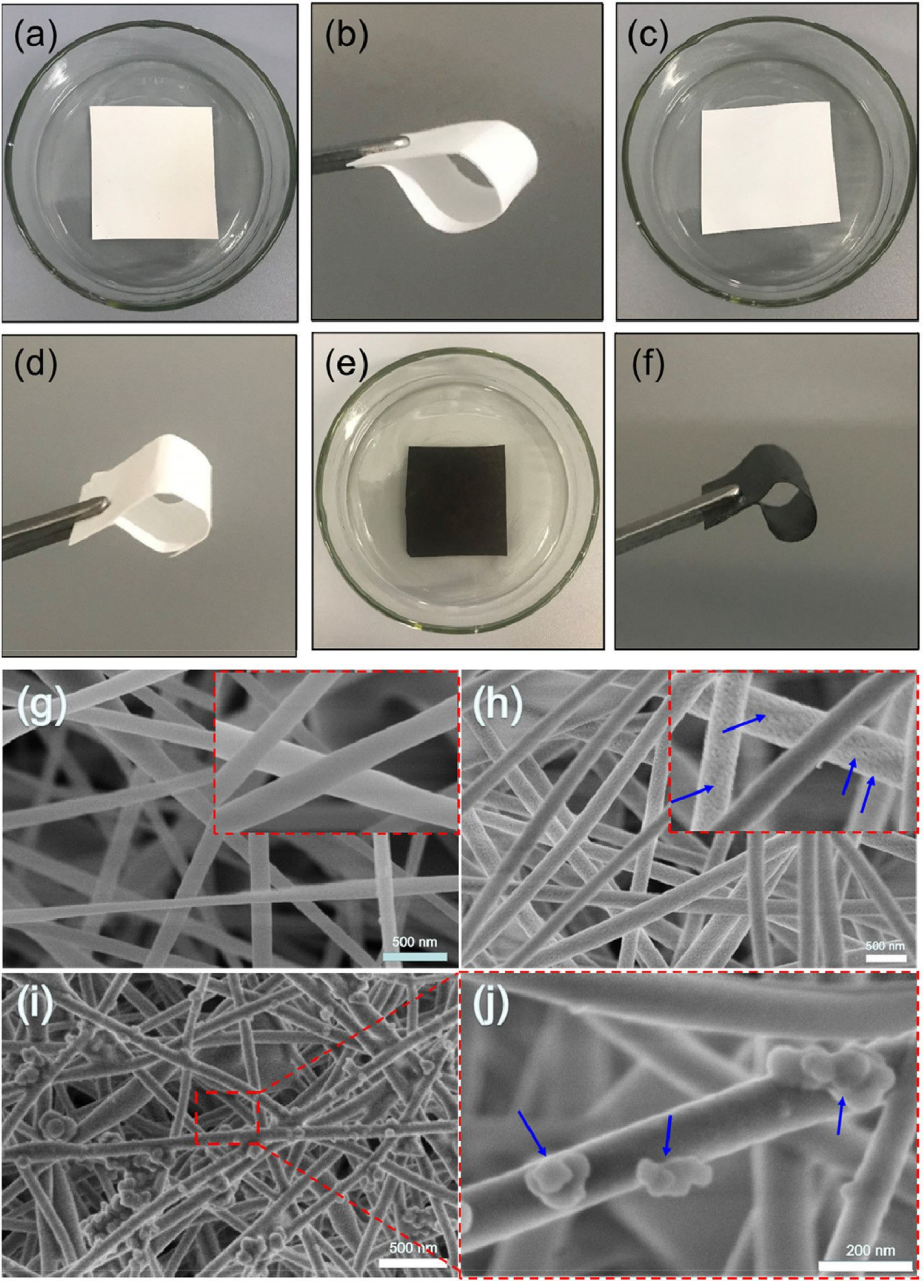
The flexibility of the as-prepared PVA/TEOS membrane (a, b), SiO_2_ nanofiber membrane (c, d) and Pd-decorated polydopamine-SiO_2_/PVA electrospinning nanofiber membrane (e, f). FE-SEM images of the as-prepared PVA/TEOS nanofiber membrane (g), SiO_2_ nanofiber membrane (h), and Pd-decorated polydopamine-SiO_2_/PVA nanofiber membrane with different magnifications (i, j). Figure 5 was reprinted from [83] Journal of Cleaner Production, vol. 275, by H. Yin; J. Zhao; Y. Li; L. Huang; H. Zhang; L. Chen, “A novel Pd decorated polydopamine-SiO_2_/PVA electrospun nanofiber membrane for highly efficient degradation of organic dyes and removal of organic chemicals and oils”, article no. 122937, Copyright (2020), with permission from Elsevier. This content is not subject to CC BY 4.0.

A novel functionalized HNT-incorporated PVDF nanofiltration membrane has been developed by Zeng et al. for the removal of organic dyes and heavy metal ions from water. The HNTs has been modified with 3-aminopropyltriethoxysilane (APTES) in order to overcome the aggregation of HNTs owing to their high length-to-diameter ratio, but it has also enhanced the separation ability of the membrane. Owing to the higher number of negative charges in the presence of A-HNTS the membrane showed 94.9% dye rejection rate for anionic direct red 23 and efficiently removed Cu^2+^, Cd^2+^, and Cr^6+^ [82]. In addition to the above, Yin and co-workers successfully fabricated a superhydrophilic flexible nanohybrid membrane of Pd NP-decorated polydopamine-SiO_2_/PVA, which can simultaneously remove organic dyes, chemicals, and oils [83]. (Figure 4 and Figure 5) Superhydrophilicity was found to be a synergistic effect of numerous hydroxy groups from SiO_2_ NPs and the nano/microscale surface roughness. The organic dyes were degraded by catalytic activity, while oil and chemicals were removed by filtration. The filtration of kerosene, hexane, petroleum ether, chloroform, and toluene each mixed with water was carried out and the removal efficiency reached a value of 99.9%, while the dye degradation efficiency for both anionic (MB) and cationic (CR) dyes had a high value of 99%. The membrane also demonstrated excellent reusability and stability and was operational even in alkaline, acidic salty, and hot water environments [83].

#### 5.2 Water purification membrane technologies

**5.2.1 Adsorption membranes.** Adsorption membranes decontaminate wastewater by adsorbing the impurities via chemisorption or physisorption. Polymeric membranes have functional groups that can interact with the solutes. In chemisorption, an irreversible chemical bond is formed between the adsorbate and the adsorbent, whereas in physisorption, only a reversible physical bond due to electrostatic interactions or intermolecular interactions forms between adsorbate and the adsorbent. An ideal adsorption membrane should exhibit both high adsorption capacity and a high adsorption rate. ENH membranes have been widely used as adsorption membranes due to the high surface area for particle adsorption. The hybridization of the electrospun membranes with additives improved the properties compared to pristine polymer membranes. GO, Fe_2_O_3_ and other metal oxides, HNTs, activated carbons, and zeolite are some of the frequently used additives for ENH membranes. The synergistic effect of both the additives and the pristine polymer results in a much better product with enhanced adsorption properties. These additives often provide more functional groups for the adsorption process, simultaneously imparting hydrophilic properties to the hydrophobic polymer membranes. The hydrophilicity of the membranes notably inhibits membrane fouling due to oils and other hydrophobic matter. For example, GO is highly hydrophilic and will impart hydrophilicity to the ENH membrane. Blending of iron oxides into ENH membranes will impart magnetic properties, which facilitate membrane removal by an external magnetic field. As summarized in Table 1, different nanomembranes have been fabricated via electrospinning and electrospraying to be used as adsorbent membranes to remove metal ions and organic dyes. The polymers used for the fabrication of adsorbant membranes along with the additive are summarized and the adsorption capacities of each ENH membranes are tabulated in Table 1.

**Table 1:** Recent advances in electrospun nanohybrid membranes as adsorption membranes in water treatment/purification.

Polymer	Additive	Remarks	Adsorption capacity	Ref

PU	GO	trifunctional, superhydrophobic antifouling membrane. Removes organic dyes, bacteria, and possess 99.99% oil separation efficiency. Good antibacterial properties for both Gram-negative and Gram-positive bacteria.	MB - 109.88 mg/g RhB - 77.15 mg/g	[84]
PVDF	GO	MB dye removal	MB - 621.1 mg/g	[85]
PEO/CS	HNT/Fe_3_O_4_	high antibacterial activity for *E. coli* and *S. aureus*. Adsorption capability: Cr^6+^ < Cd^2+^ < Cu^2+^ < Pb^2+^	Cr^6+^ - 67.024 mg/g	[86]
PEO/CS	Fe oxides	out of Fe, Zr and Cu oxides incorporated composites Fe oxide incorporated membrane showed the best adsorption for As^3+^	As^3+^ - 31.6 mg/g	[87]
CS/PEO	activated carbon	heavy metal ion adsorption	Cr^6+^ - 261.1 mg/g Fe^3+^ - 217.4 mg/g Cu^2+^ - 195.3 mg/g Zn^2+^ - 186.2 mg/g Pb^2+^ - 176.9 mg/g	[88]
PES	V_2_O_5_ NPs	MB dye adsorption; the effect of temperature and pH value on adsorption has been studied. Highest adsorption capacity of 85% in basic conditions and high temperature.		[89]
CS/PVA	Zeolite	adsorption of MO dye	153 mg/g	[90]
CS/PVA	Zeolite	heavy metal adsorption	Cr^6+^ - 8.84 mg/g Fe^3+^ - 6.14 mg/g Ni^2+^ - 17.61 mg/g	[91]
PVA	lignin	adsorption of Safranine T dye	140.3 mg/g	[92]
PVA	hollow α-Fe_2_O_3_	MO dye removal	80.6 mg/g	[93]
PVA	polyphosphoric acid-modified MMT	adsorption of organic dye; removal of more than 90% of the organic dyes within 10 min	MB - 293.9 mg/g RdB - 244.77 mg/g Rose Bengal - 296.13 mg/g	[94]
PAN	EDTA-intercalated LDH	adsorption of Cu^2+^; prevents loss and aggregation of adsorbents	Cu^2+^ - 120.77 mg/g	[95]
PAN	polydopamine, MnO_2_	adsorption of Pb^2+^	Pb^2+^ - 185.19 mg/g	[96]
PS	electrosprayed nanospheres of layered silicate rectorite mixed with CS	adsorption of Cu^2+^	Cu^2+^ - 122.46 mg/g	[97]

**5.2.2 Filtration membranes.** The removal of particulate matter via filtration membranes is mainly pressure driven or osmotically driven and has two operating modes, namely dead-end filtration and crossflow filtration. In dead-end filtration, the feed is applied perpendicular to the membrane filter while in crossflow filtration, the feed is applied along the membrane. The following section will present recently developed electrospun nanohybrid membranes used as filtration membranes. The high porosity, interconnected pore structures, and the easy manipulation of membrane morphology and mechanical strength has made electrospun membranes a viable substitute for conventional membranes. Most of the membrane development researches have focused on the alleviation of fouling of the membranes and on increasing flux and wettability by the incorporation of fillers.

**5.2.2.1 Pressure-driven filtration.** Pressure-driven filtration utilizes the transmembrane pressure difference as the driving force for membrane permeation. Pressure-driven filtration is classified into MF, ultrafiltration (UF), nanofiltration (NF), and reverse osmosis (RO), depending on the membrane pore sizes and the applied pressure. A low pressure is applied for the highly porous membranes with large pores and the applied pressure increases with the decrease of the pore size.

MF membranes have pore diameters in the range of 0.1–5 μm and require a pressure typically below 1 bar. Particles with dimensions greater than 0.1 μm will be rejected while smaller particles are allowed to permeate through the membrane. MF filters have the potential to filter out suspended particles, major pathogens, large bacteria, proteins, and yeast cells from aqueous media [98].

UF membranes have pore diameters of 0.01–0.1 μm and a pressure of 1–10 bar is applied for the filtration. UF membranes are reported to have a high removal rate of turbidity, organic matters, parasites, and viruses as well [99].

NF membranes lie in the lower range of UF membranes and upper range of RO filtration membranes. Usually, the pore size for NF is defined by a molecular weight cutoff, which is 100–1000 Da. Studies have shown that the combined effect of steric, Gibbs–Donnan, and dielectric effects results in salt rejection in NF [100].

Table 2 summarizes the recently developed electrospun MF, UF, and NF nanohybrid membranes for water filtration. The different polymers along with the additives used for the ENH membranes are tabulated. In addition, the water flux and the rejection rate of the specific pollutants obtained with the membranes are tabulated.

**Table 2:** Recently fabricated electrospun nanohybrid membranes for microfiltration, ultrafiltration, and nanofiltration.

Filtration	Polymer	Additive	Water flux	Rejection rate	Pressure	Ref

MF	PAN	*para*-aminobenzoate, alumoxane NPs	2120 LMH		0.1 bar	[101]
	PAN	polycitrate-*para*-aminobenzoate alumoxane NPs	1932 LMH		0.1 bar	[102]
	aminated PAN	GO	10000 LMH	≥98% oil–water emulsions		[103]
	PVAc/N6	SiO_2_	4814 LMH/bar	99% oil	0.28 bar	[104]
	PVDF	GO	800 LMH	99% 0.1 mg/L kaolin solution	1 bar	[105]
	CA	chitin nanocrystals	14217 LMH		0.5 bar	[106]

UF	PVDF/CS	UiO-66 NH_2_ MOF NPs, ZIF-8 MOF NPs	470 LMH	98.1% - BSA 95.6% - Cr^6+^	1 bar	[107]
	PES	hydrous manganese dioxide	4263 LMH	92.5–95% oil		[108]

NF	PHB/calcium alginate hydrogel	CNT	68.61 to 150.72 LMH	90% (dyes >600 g/mol MW) 99.57% BSA	1 to 7 bar	[109]
	PSF	keratin powder	2000 LMH	76% dyes	2.07 bar	[110]

RO is extensively used in water purification and water desalination as it can remove even the smallest pollutants. Semipermeable membranes reject the dissolved constituents based on size exclusion, charge exclusion, and physical/chemical interactions between the solvent, solute, and the membrane. RO membranes can either be asymmetric with only a single polymer or composites with two or more polymers [111]. Currently, thin film nanocomposites (TFC) are employed as RO membranes. TFCs consist of a porous substrate layer on which a highly cross-linked active polymer layer is deposited via interfacial polymerization. It was believed that the substrate layer of the RO membrane has an indirect effect on the RO membrane performance. Recent studies showed, however, that the pore size and the hydrophilicity of the substrate layer have a significant impact on the performance of the RO filters by allowing the formation of a thin active barrier layer on the substrate [112]. Recently, Wang et al. reported a TFC RO membrane of cellulose nanofiber-modified electrospun PAN on a nonwoven PET support. It allowed for the formation of a uniform barrier layer with a higher permeation flux than the substrate membrane without cellulose nanofibers. The optimized RO membrane exhibited a rejection rate of 96.5% against NaCl (500 ppm) and a flux of 28.6 LMH at 0.7 MPa, approaching the performance of a high-flux commercial RO membrane (DOW FILMTEC™ XLE) [113]. Though there have been several studies on electrospun membranes used for RO filtration, studies on electrospun hybrid membranes with incorporated additives are very scarce. Futher research needs to be initiated in order to improve the performance in RO filtration by utilizing hybrid electrospun membranes.

**5.2.2.2 Osmotically driven filtration.** Forward osmosis (FO) is the filtration mechanism in which the water from the feed solution is filtered out into a draw solution through osmotic pressure. The draw solution is mostly a salt. FO membranes also have the TFC form. The major drawbacks of the FO membranes are fouling and internal concentration polarization (ICP), which deteriorate the effective osmotic driving force. Biofouling has been alleviated by the incorporation of fillers with antibacterial properties such as Ag NPs. The fouling due to hydrophobic organic matter, such as proteins and oils, has been alleviated by enhancing the hydrophilicity of the membranes though the incorporation of hydrophilic inorganic particles. As previous studies have been reported ICP is directly related to the structure parameter (*S*) of the substrate. Low thickness, high porosity, and low tortuosity are the most favourable substrate morphologies to build up active FO membranes, which are mostly highly crosslinked PA layers [114]. Electrospun nanofiber membranes have been widely used in the fabrication of substrates for TFC FO filters due to the ultralow structure parameters, which will minimize ICP. The electrospun membranes have been modified with many fillers such as Ag NPs, TIO_2_ NPs, GO, SiO_2_ NPs and carbon nanotubes (CNTs), and with combinations of these as shown in the Table 3. ENH membranes have shown enhanced water fluxes while mitigating the above mentioned drawbacks and also have shown better mechanical properties.

**Table 3:** Recently developed electrospun nanohybrid membranes for forward osmosis.

Polymer	Modifier NP	*S* (μm)	Osmotic water flux (LMH)		Reverse salt flux (*J*_s_/*J*_w_)		Ref

			AL-FW^a^	AL-DS^b^	AL-FW	AL-DS	

PAN	CNT		49.2	61.6	7.2 gMH	7.7 gMH	[114]
PAN	AG NPS		21.58	29.21			[115]
PEI^c^	SiO_2_ NPs	174	42	72	0.25 g/L		[116]
PSF	TiO_2_ NPs		55	65	15 gMH	20 gMH	[117]
PVDF	SiO_2_ NPs	29.7	83		0.77 gMH		[118]
PVDF	GO	85.5	80.9		0.42 g/L		[119]

^a^Active layer facing feed solution; ^b^active layer facing draw solution; ^c^polyetherimide.

**5.2.3 Membrane distillation.** MD is a hydrophobic membrane-based thermal process, which is used in water treatment. Only water vapor of the feed solution is passed through the filter membrane pores leaving the nonvolatile matter in the feed at a relatively low temperature and pressure [120]. The water vapor diffuses through the membrane due to the temperature difference between the two sides of the membrane. The process operates at very low temperatures such that it can use waste heat [121] or even solar energy [122], and geothermal energy [123]. It is extensively used in desalination and wastewater treatment with theoretically 100% rejection of solute to produce high-quality water without any pretreatment. Compared to other membrane-based techniques, such as NF and RO, MD has many technical advantages in producing high water recovery with less energy consumption. Nevertheless, the membrane has its own drawbacks, such as low flux and wetting of the pore interiors, which will reduce its efficiency over time. To overcome those downsides of the MD process, ENH membranes have gained a great attention.

**Figure 6:**
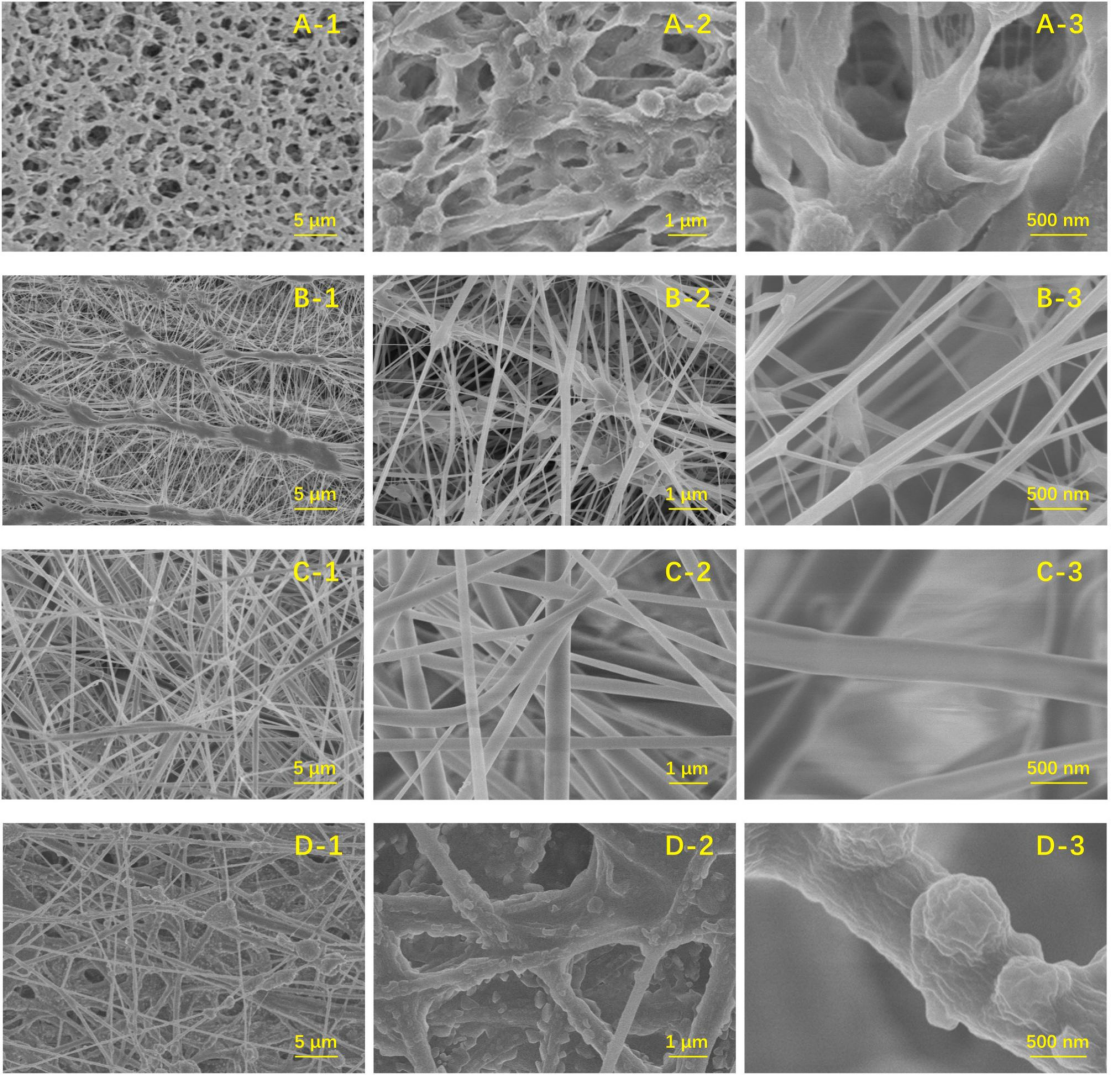
SEM images of the surfaces of (A) the commercial PVDF membrane, (B) the commercial PTFE membrane, (C) the CA-PDTS fibrous membrane, and (D) the CA/SiNPs-PDTS fibrous membrane. Figure 6 was reprinted from [125], Desalination, vol. 468, by D. Hou; C. Ding; C. Fu; D. Wang; C. Zhao; J. Wang, “Electrospun nanofibrous omniphobic membrane for anti-surfactant-wetting membrane distillation desalination”, article no. 114068, Copyright (2019), with permission from Elsevier. This content is not subject to CC BY 4.0.

**Figure 7:**
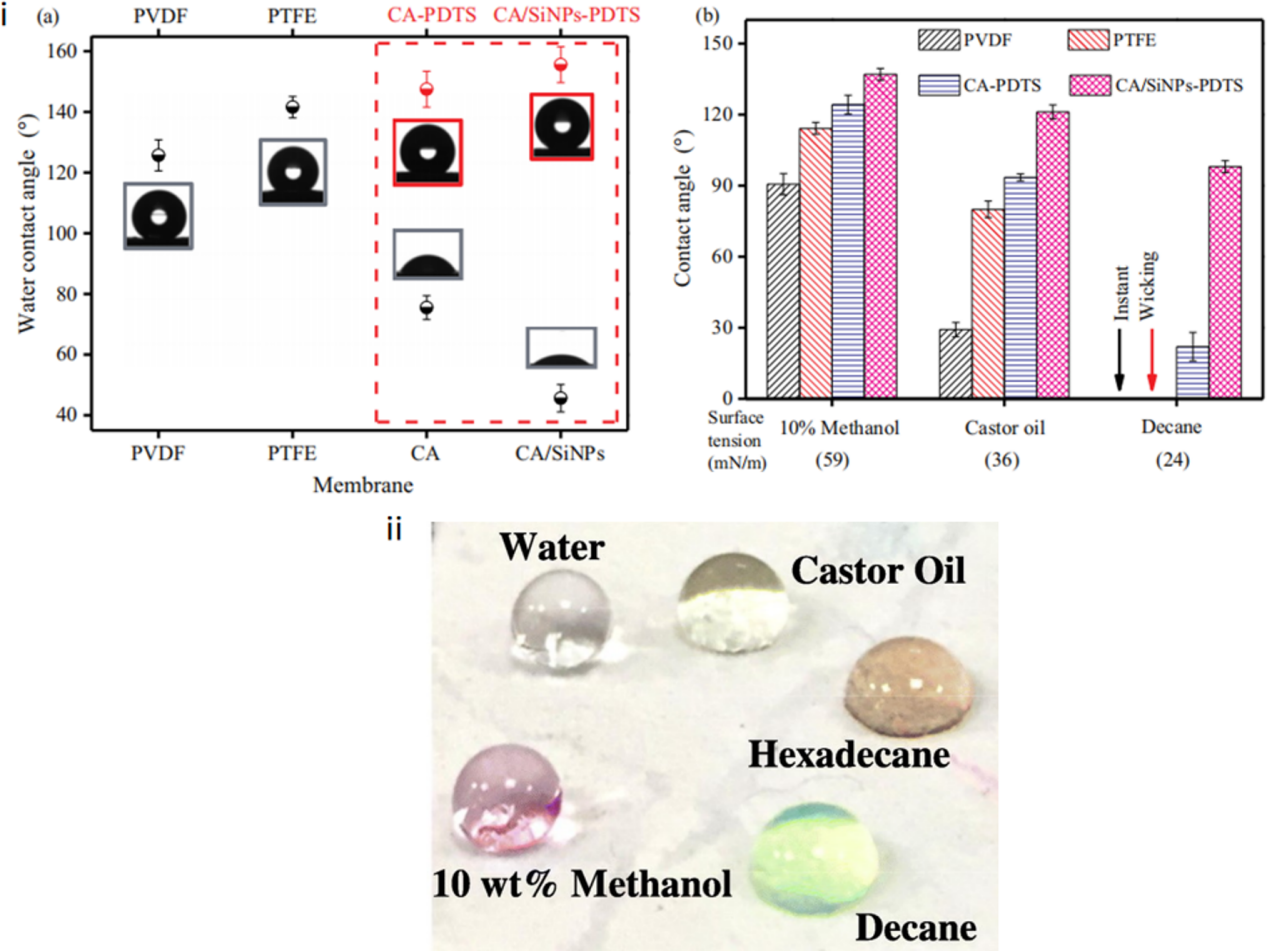
(i) (a) Water contact angles of the commercial PVDF and PTFE hydrophobic membranes and the fabricated fibrous membranes; (b) comparison of contact angles among the commercial PVDF and PTFE hydrophobic membranes and the fabricated fibrous membranes with different liquids. (ii) Photos of different liquids on the omniphobic membrane. Figure 7 was reprinted from [125], Desalination, vol. 468, by D. Hou; C. Ding; C. Fu; D. Wang; C. Zhao; J. Wang, “Electrospun nanofibrous omniphobic membrane for anti-surfactant-wetting membrane distillation desalination”, article no. 114068, Copyright (2019), with permission from Elsevier. This content is not subject to CC BY 4.0.

For example, Hou et al. synthesized a PVDF-HFP/SiNPs electrospun hybrid membrane for direct-contact MD (DCMD). The nanoscale roughness imparted by the hydrophobic fumed SiNPs made the surface superhydrophobic and the membrane showed a water contact angle greater than 150°. Additionally, the membrane yielded a maximum flux of 48.6 LMH with 99.99% rejection of NaCl at a feed temperature of 80 °C [124]. The same group later developed a novel omniphobic membrane for anti-surfactant-wetting MD with surface-fluorinated CA/SiNPs, which exhibited superior anti-wetting properties compared with commercial PVDF and PTFE membranes, with a water contact angle of 155.6° and with a contact angle of 95.3° for decane [125]. (Figure 6 and Figure 7). Dong et al. developed a fluoroalkyl silane (FAS)-grafted glutaraldehyde-crosslinked PVA membrane with low surface energy and a water contact angle of 158° for vacuum MD (VMD). The membrane demonstrated a high and stable permeate flux of 25.2 LMH, which is 70% higher than the commercial PTFE membranes. Also, the membrane showed great potential to be used in desalination and in the removal of volatile organic compounds [126]. Huang et al. also developed a coaxially electrospun, surface-fluorinated PVA-based membrane with incorporated SiNPs, which demonstrated superamphiphobicity, for anti-surface wetting MD. A water contact angle of 154.2° and a mineral oil contact angle of 149.0°, both greater than the values for commercial PVDF membranes, were observed for the prepared membrane [127]. In another study, Lee et al. developed a 1*H*,1*H*,2*H*,2*H*-perfluorooctyltriethoxysilane (FTES)-functionalized TiO_2_-incorporated PVDF-HFP-based hydrophobic membrane with a maximum water contact angle of 149° and a flux of 40 LMH without any noticeable decrease in the permeability even after operating for seven days [128]. Liao et al. also studied the potential use of superhydrophobic fluorinated silica-PVDF in DCMD and found that the membrane showed a water contact angle of 150°. Also, the membrane has exhibited a flux greater than 18 LMH, which is greater than that of the commercial PVDF flat sheets (10 LMH) [129]. Prince et al. reported a PVDF–clay nanocomposite nanofiber membrane used as a hydrophobic membrane in DCMD with 99% salt rejection and a maximum water contact angle of 154.20° [130]. Yang et al. also developed a membrane for desalination of water via DCMD by incorporating MOF (Iron-BTC) into superhydrophobic PVDF. The membrane exhibited a water contact angle of 138.06° with a flux of 2.87 kg/(m^2^·h) of water vapor and 99.99% NaCl rejection. The temperatures of feed and permeate temperature were 48 °C and 16 °C, respectively [131]. A hydrophobic membrane for VMD by incorporating hydrophobic FTES-functionalized GO into PVDF electrospun over a nonwoven PP fabric has been developed by Li et al. for improved desalination. A water contact angle of 140.5°, with a maximum water vapor permeation flux of 36.4 kg/(m^2^·h) and a salt rejection value greater than 99.9% at a temperature of 50 °C were exhibited by the membrane [132]. Recently, Elmarghany et al. fabricated a triple-layer nanocomposite membrane by electrospinning incorporating CNT as the additive to use in direct-contact MD. To obtain a high porosity, a polyethersulfone (PES)/CNT hybrid was used as the mid layer and PVDF-HFP/CNTs was deposited on the outer and inner surfaces of the membrane by electrospinning to get a noticeably hydrophobic surface with a water contact angle of 144°. The membrane exhibited a maximum permeation flux of 22.2 LMH with a salt rejection of 99.3% at a feed temperature of 65 °C [133].

**5.2.4 Photocatalytic membranes.** The removal of many organic pollutants from wastewater by converting them into less harmful and more biodegradable products using advanced oxidation processing (AOP) based on photocatalytic oxidation is a fascinating technique. Applicability to a wide variety of contaminants, environmental compatibility, and mild oxidation conditions are some of the benefits of using photocatalytic active materials in water purification [134]. A photocatalyst is a semiconductor with a wide bandgap, which is photoactive in visible and/or near-UV light. To be used in water purification it should be biologically and chemically inert while being inexpensive and nontoxic [135–136]. When photocatalysts are irradiated with light, the electrons of the filled valence band are promoted to the empty conduction band, leaving a hole in the valence band. These electrons will either recombine with holes or the electron–hole pair will interact individually with other molecules in the solution. Radicals are produced when the holes react with electron donors in the solution [137].

The photocatalysts are usually dispersed in contaminated water, which makes it difficult to recover the photocatalysts after purification and requires additional post-purification steps. In recent years, to overcome this issue, immobilization of photocatalysts onto polymeric substrates has been introduced [138]. Electrospun porous polymer membranes with high surface area and high permeability have gained the attention of researchers as supports for the photocatalysts. Extensive research has been reported on the use of various electrospun polymers incorporated with photocatalytic semiconductor nanoparticles.

Owing to the chemical stability, low cost, acceptable bandwidth, and eco-friendliness, TiO_2_ has become the most extensively used photocatalyst in water remediation [139]. For instance, Bode-Aluko et al. developed a superhydrophobic antifouling photocatalytic membrane by incorporating TiO_2_ into PAN, which exhibited 99% photodegradation of MB dye within 3 h and also hindered the growth of bacteria, having a greater effect on the Gram-positive bacterium, *Bacillus sp,* under simulated visible light of 1000 W/m^2^ [140]*.* Blanco et al. also developed a TiO_2_-doped PA 6 nanofiber membrane, which decreased 70% of the organic pollutant model, remazol black B dye under UV light after 240 min and removed *Escherichia coli* and other coliform bacteria successfully in 24 h of contact [141]. Wu et al. developed a flexible and porous electrospun fiber mat incorporating TiO_2_ and SiO_2_ in PVP followed by carbonization to produce a TiO_2_/SiO_2_/carbon electrospun nanofiber mat for the degradation of RhB and 4-nitrophenol [142]. The same group has also developed a multiwall carbon nanotubes (MWCNTs)/Ag_3_PO_4_/PAN ternary composite fiber membrane that showed photocatalytic activity towards RhB under visible light irradiation [143]. In another study, Salazar et al. studied the incorporation of TiO_2_ nanoparticles, surface modified with Ag nanoparticles into PVDF-HFP and observed that the membrane has photocatalytic ability along with antimicrobial activity [144]. Moreover, Yar et al. developed a TiO_2_/ZnO nanoparticle-embedded electrospun PAN hybrid membrane that degraded 3 mL of 1.0 × 10^−5^ M malachite green dye completely in 240 min under UV irradiation [145]. Li et al. also reported on a CS/g-C_3_N_4_/TiO_2_ electrospun fiber mat for the efficient removal of Cr^6+^ by adsorption and photocatalysis. The synergistic effect has noticeably improved the removal efficiency of Cr^6+^ by 50% compared to pure adsorption under visible light [146]. ZnO, being a wide-bandgap semiconductor, absorbs a larger fraction of the solar spectrum than TiO_2_, which makes it an alternative with higher efficiency than TiO_2_. Ognibene et al. fabricated a photocatalytic membrane with ingrown ZnO nanorods in PES that showed a 78% degradation of methylene blue dye under UV light irradiation [137]. Campagnolo et al. also developed an Au/ZnO-based nanoporous polymethylmethacrylate (PMMA) electrospun membrane [147], while Rosman et al. electrospun a fibrous photocatalytic mat with embedded ZnO/Ag_2_CO_3_/Ag_2_O in PVDF, which exhibited a 99.62% photodegradation of reactive red 120 (RR120) in 300 min [148]. Kanjwal et al. developed a NiO/ZnO–PVA composite and for the photocatalytic removal of dairy effluents and MB dye under visible light irradiation. A maximum degradation of 80% for dairy effluents in 23 h and of 100% for MB in 90 min was achieved by the membrane [149]. Chen et al. fabricated a GO/ZnS-CNFs membrane with electrospun PAN, which exhibited excellent photocatalytic degradation of *p*-aminotoluene and phenol under mild conditions [150]. Moreover, Zhong et al. developed flexible membranes from electrospun carbon nanofiber/tin(IV) sulfide (CNF@SnS_2_) core/sheath fibers to treat Cr^6+^-contaminated water and obtained a complete degradation of a 250 mg/L aqueous Cr^6+^ solution within 90 min. Additionally, the membrane had clear advantages such as flexibility and a self-supporting porous architecture with structural stability over SnS_2_ nanoflowers [151]. In another report, Dai et al. demonstrated a zeolitic imidazole framework(ZIF)/GO/PLA electrospun hybrid for the removal of organic dye from contaminated water and observed that the membrane has the ability to remove 90% of methylene blue with a minimal concentration of ZIF/GO [3].

**5.2.5 Bactericidal membranes.** Bacterial pathogens are responsible for many water-borne diseases that threaten human health, such as giardiasis, gastroenteritis, cholera, and cryptosporidiosis. Although there are many traditionally used simple and effective chemical agents, such as chlorine and related compounds, for water disinfection, they have drawbacks that need to be overcome using an alternative. These traditional chemicals take a long time to inactivate bacterial pathogens, consume a significant amount of chemicals in the disinfecting process and most importantly, produce harmful byproducts in the process [152]. Broad-range antimicrobial activity in a short period of time, health safety, affordability, hydrophilicity, and no production of toxic byproducts during the process are the properties of an ideal water disinfection [153]. Electrospun membranes with adjustable pore size and high porosity will enable the filtration of pathogen cells, while the incorporation of bactericidal matter by facile modification processes will inhibit bacterial cell growth. There are many ENH membranes fabricated that have been used as bactericidal membranes, which showed potentials to be used in water disinfection in disaster-affected areas.

An antibacterial membrane has been fabricated by Parekh et al. by surface coating an electrospun PAN membrane with Ag NPs to be used in water remediation. The membrane has shown 100% reduction of Gram-negative bacteria with a filtration rate of 8.0 mL/cm^2^ min [154]. Shalaby et al. developed an antibacterial membrane by electrospinning PAN blended with Ag, CuO, or ZnO nanoparticles as bactericides and observed that the membranes with CuO or ZnO NPs showed higher antibacterial activity towards *E.coli* than towards *S.aureus* [155]. In another work, He et al. fabricated a La(OH)_3_ nanorod/PAN composite by electrospinning followed by an in situ precipitation process to obtain a membrane for fast filtration to remove cells and also removes phosphates from an aqueous medium. The introduction of La(OH)_3_ nanorods resulted in a positively charged membrane surface with reduced pore sizes and higher mechanical strength, which led to high bacterial cell retention during microfiltration. The fast and efficient binding of phosphates with lanthanum resulted in 97% removal of phosphate groups, which will make the bacteria undergo nutrient starvation and will prevent the recontamination of water [156]. Yang et al. also demonstrated a CS/PVA/GO electrospun hybrid membrane that exhibited antibacterial activity against both Gram-positive and Gram-negative bacteria [157].

ENH membranes have been used as disinfectant membranes in point-of-use water treatment as well. A novel gravity-driven nanofibrous membrane has been electrospun by Wang et al. to be used in point-of-use filters as a disinfectant. The authors coated an electrospun porous nanofibrous PAN substrate with polydopamine and used the reduction ability of polydopamine for the in situ preparation of Ag NPs [158]. The membrane achieved a maximum water flux of 130 LMH, showing its potential to process solely by gravity filtration, eliminating the need of electrical power and exhibited a significant disinfecting capability with its strong antibacterial activity and high physical rejection. The permeate was completely free of viable bacteria and the authors suggested that this membrane will be ideal for the water disinfection in disaster-affected areas [158]. Xie et al. also developed a single-walled carbon nanotubes (SWNTs)-incorporated PAN/polyurethane/polyaniline hybrid membrane by co-electrospinning for point-of-use water treatment. The membrane exhibited a complete removal of bacteria in the absence of electrolysis by sieving. With electrolysis along with filtration, the membrane achieved a significant enhancement of bacterial inactivation. And it is tested that the excessive release of SWNTs is restricted by the composite, which will result in long-lasting disinfectant properties of the membrane [159].

### 6 Future potential of electrospun fiber membranes in water purification

Electrospinning as a versatile, straightforward technique has been exploited in many areas for the preparation of nanofiber membranes. The significant porosity, interconnected pores with a narrow distribution of pore sizes, high surface area, superior mechanical and chemical properties, flexibility, easy fabrication, and cost-effectiveness are major advantages of electrospun membranes as discussed in the above sections. Further, electrospinning allows for a facile incorporation of functional nanomaterials into the fabricated membranes, which leads the path to have control over the surface chemistry of the electrospun membranes.

In addition to the above discussed water treatment processes, novel improvements should be explored for ENH membranes in water remediation. For instance, multifunctional hybrid electrospun membranes can be developed by the convergence of properties of several nanomaterials. These membranes will be capable of disinfecting water, decontaminating, and separating pollutants in one step. These multifunctional hybrid membranes will be ideal for efficient point-of-use water treatments. Further, the facile blending of nanomaterials into ENH membranes can allow for the incorporation of sensors into the membrane for the detection and real time tracking of the removal of the pollutants such that users can easily recognize the time for recycle the filtering device.

Although ENH membranes have shown many superior properties, there are some constrictions regarding which further studies are required. For instance, it is hard to obtain submicrometer pore diameters and fiber diameters. Hence more studies have to be conducted in order to obtain ultrafine pores and fibers, which will increase the efficiency of eliminating ultrafine particulate matter in wastewater. Correspondingly, the use of more biodegradable polymers, the increase of antifouling properties, and more importantly, the lifetime and the physicochemical durability of the ENH membranes are some of the areas that need further research. Moreover, the wetting abilities of the ENH membrane surfaces have to be modified concerning the actual use of the membrane. Advanced research has to be conducted in order to increase the superhydrophobicity, superhydrophilicity, and most importantly, the omniphobicity, which is beneficial in the membrane distillation process. Another critical issue of electrospun membranes arises when moving to the production at an industrial scale. Hence, for ENH membranes to be far-reaching and allowing for access to safe water resources to larger groups of people the research has to be steered to enhance the mass production and thereby commercialization of ENH membranes.

## Conclusion

In this review, we have first given an insight into the versatility of ENH membranes in different fields in which they are already utilized, followed by an elaborate discussion on the electrospinning technique with its limiting factors for the control of membrane morphology with the variation of parameters. Next, the advantageous use of the electrospun membranes over conventional membranes in water purification has been discussed. Then, we have detailed the recently developed ENH membranes in solving global water issues. A description of the use of ENH membranes in removing various common water pollutants, such as heavy metal ions, radioactive metal cations, oils, organic matter, textile dyes, and biological pollutants, through adsorption, filtration, photocatalytic, and bactericidal capabilities of the hybrid membranes has been given. Finally, a perspective has been provided on the future research paths to fill the gaps of the field and to enhance the properties of the existing water remediation ENH membranes.

In conclusion, electrospinning provides an excellent platform for the development of efficient water treatment materials and has seen significant progress in the past few years to become the next generation of filter media. They have shown many advantageous properties such as high surface area, high porosity, facile functionalization, high water permeability, low energy consumption and less fouling, compared to conventional membranes used in water purification. However there are still many drawbacks that have to be overcome before reaching industrial scale appliciation. Further efforts are expected to solve these difficulties and stimulate the tremendous growth of novel ENH membranes for water treatment.

## References

[1] Lalia B S, Kochkodan V, Hashaikeh R, Hilal N (2013). Desalination.

[2] Ray S S, Chen S-S, Li C-W, Nguyen N C, Nguyen H T (2016). RSC Adv.

[3] Dai X, Li X, Zhang M, Xie J, Wang X (2018). ACS Omega.

[4] Qiao Z, Shen M, Xiao Y, Zhu M, Mignani S, Majoral J-P, Shi X (2018). Coord Chem Rev.

[5] Feng C, Khulbe K C, Matsuura T, Tabe S, Ismail A F (2013). Sep Purif Technol.

[6] Esfahani H, Jose R, Ramakrishna S (2017). Materials.

[7] Suja P S, Reshmi C R, Sagitha P, Sujith A (2017). Polym Rev (Philadelphia, PA, U S).

[8] Giuri D, Barbalinardo M, Sotgiu G, Zamboni R, Nocchetti M, Donnadio A, Corticelli F, Valle F, Gennari C G M, Selmin F (2019). Nanoscale.

[9] Patel D K, Dutta S D, Hexiu J, Ganguly K, Lim K-T (2020). Int J Biol Macromol.

[10] Munaweera I, Aliev A, Balkus K J (2014). ACS Appl Mater Interfaces.

[11] Munaweera I, Levesque-Bishop D, Shi Y, Di Pasqua A J, Balkus K J (2014). ACS Appl Mater Interfaces.

[12] Bugatti V, Vertuccio L, Viscusi G, Gorrasi G (2018). Nanomaterials.

[13] Zhang S, Li D, Kang J, Ma G, Liu Y (2018). J Appl Polym Sci.

[14] Zhang J, Xiang Y, Jamil M I, Lu J, Zhang Q, Zhan X, Chen F (2018). J Membr Sci.

[15] Savva I, Kalogirou A S, Achilleos M, Vasile E, Koutentis P A, Krasia-Christoforou T (2016). Molecules.

[16] Ge J C, Choi N J (2017). Nanomaterials.

[17] Al-Attabi R, Morsi Y, Kujawski W, Kong L, Schütz J A, Dumée L F (2019). Sep Purif Technol.

[18] Li W-J, Shanti R M, Tuan R S (2007). Electrospinning Technology for Nanofibrous Scaffolds in Tissue Engineering.

[19] Liao Y, Loh C-H, Tian M, Wang R, Fane A G (2018). Prog Polym Sci.

[20] Alghoraibi I, Alomari S, Ahmed Barhoum M B, Makhlouf A S H (2019). Different methods for nanofiber design and fabrication. Handbook of nanofibers.

[21] Demir M M, Yilgor I, Yilgor E, Erman B (2002). Polymer.

[22] Huan S, Liu G, Han G, Cheng W, Fu Z, Wu Q, Wang Q (2015). Materials.

[23] Liu Z, Ju K, Wang Z, Li W, Ke H, He J (2019). Nanoscale Res Lett.

[24] Kim J-H, Lee J-H, Kim J-Y, Kim S S (2018). Appl Sci.

[25] Nurwaha D, Han W, Wang X (2013). J Text Inst.

[26] Megelski S, Stephens J S, Chase D B, Rabolt J F (2002). Macromolecules.

[27] Teo W E, Ramakrishna S (2006). Nanotechnology.

[28] Ren L-F, Xia F, Shao J, Zhang X, Li J (2017). Desalination.

[29] Sencadas V, Correia D M, Areias A, Botelho G, Fonseca A M, Neves I C, Gomez Ribelles J L, Lanceros Mendez S (2012). Carbohydr Polym.

[30] Macossay J, Marruffo A, Rincon R, Eubanks T, Kuang A (2007). Polym Adv Technol.

[31] Sadat-Shojai M (2016). J Mater Sci Technol.

[32] Subrahmanya T M, Arshad A B, Lin P T, Widakdo J, Makari H K, Austria H F M, Hu C-C, Lai J-Y, Hung W-S (2021). RSC Adv.

[33] Deitzel J M, Kleinmeyer J, Harris D, Beck Tan N C (2001). Polymer.

[34] Fong H, Chun I, Reneker D H (1999). Polymer.

[35] Nezarati R M, Eifert M B, Cosgriff-Hernandez E (2013). Tissue Eng, Part C.

[36] Lasprilla-Botero J, Álvarez-Láinez M, Lagaron J M (2018). Mater Today Commun.

[37] Lin J, Ding B, Yu J (2010). ACS Appl Mater Interfaces.

[38] Putti M, Simonet M, Solberg R, Peters G W M (2015). Polymer.

[39] Touny A H, Lawrence J G, Jones A D, Bhaduri S B (2010). J Mater Res.

[40] Angammana C J, Jayaram S H (2011). IEEE Trans Ind Appl.

[41] Uyar T, Besenbacher F (2008). Polymer.

[42] Pelipenko J, Kristl J, Janković B, Baumgartner S, Kocbek P (2013). Int J Pharm.

[43] De Vrieze S, Van Camp T, Nelvig A, Hagström B, Westbroek P, De Clerck K (2009). J Mater Sci.

[44] Huang L, Bui N-N, Manickam S S, McCutcheon J R (2011). J Polym Sci, Part B: Polym Phys.

[45] Medeiros E S, Mattoso L H C, Offeman R D, Wood D F, Orts W J (2008). Can J Chem.

[46] Lu P, Xia Y (2013). Langmuir.

[47] Pakravan M, Heuzey M-C, Ajji A (2011). Polymer.

[48] Lonbani M P (2012). Production of chitosan-based non-woven membranes using the electrospinning process.

[49] Kaur S, Sundarrajan S, Rana D, Sridhar R, Gopal R, Matsuura T, Ramakrishna S (2014). J Mater Sci.

[50] Essalhi M, Khayet M (2014). J Membr Sci.

[51] Ravanchi M T, Kargari A (2009). New Advances in Membrane Technology. Advanced Technologies.

[52] Van Der Bruggen B, Vandecasteele C, Van Gestel T, Doyen W, Leysen R (2003). Environ Prog.

[53] Wang X, Hsiao B S (2016). Curr Opin Chem Eng.

[54] Tai M H, Gao P, Tan B Y L, Sun D D, Leckie J O (2014). ACS Appl Mater Interfaces.

[55] Wang R, Zhang L, Chen B, Zhu X (2020). J Membr Sci.

[56] Su Q, Zhang J, Zhang L-Z (2020). Desalination.

[57] Kim H-C, Choi B G, Noh J, Song K G, Lee S-h, Maeng S K (2014). Desalination.

[58] Feng Q, Wu D, Zhao Y, Wei A, Wei Q, Fong H (2018). J Hazard Mater.

[59] Brandes R, Belosinschi D, Brouillette F, Chabot B (2019). J Environ Chem Eng.

[60] Surgutskaia N S, Martino A D, Zednik J, Ozaltin K, Lovecká L, Bergerová E D, Kimmer D, Svoboda J, Sedlarik V (2020). Sep Purif Technol.

[61] Li L, Wang F, Lv Y, Liu J, Bian H, Wang W, Li Y, Shao Z (2018). ACS Omega.

[62] Yari S, Abbasizadeh S, Mousavi S E, Moghaddam M S, Moghaddam A Z (2015). Process Saf Environ Prot.

[63] Talebi M, Abbasizadeh S, Keshtkar A R (2017). Process Saf Environ Prot.

[64] Aliahmadipoor P, Ghazanfari D, Gohari R J, Akhgar M R (2020). RSC Adv.

[65] Ivshina I B, Kuyukina M S, Krivoruchko A V, Elkin A A, Makarov S O, Cunningham C J, Peshkur T A, Atlas R M, Philp J C (2015). Environ Sci: Processes Impacts.

[66] Peng Y, Guo Z (2016). J Mater Chem A.

[67] Obaid M, Barakat N A M, Fadali O A, Motlak M, Almajid A A, Khalil K A (2015). Chem Eng J.

[68] Zhang P, Tian R, Lv R, Na B, Liu Q (2015). Chem Eng J.

[69] Ge J, Zhang J, Wang F, Li Z, Yu J, Ding B (2017). J Mater Chem A.

[70] Shang Y, Si Y, Raza A, Yang L, Mao X, Ding B, Yu J (2012). Nanoscale.

[71] Ma W, Zhang Q, Samal S K, Wang F, Gao B, Pan H, Xu H, Yao J, Zhan X, De Smedt S C (2016). RSC Adv.

[72] Jiang Z, Tijing L D, Amarjargal A, Park C H, An K-J, Shon H K, Kim C S (2015). Composites, Part B.

[73] Reshmi C R, Sundaran S P, Juraij A, Athiyanathil S (2017). RSC Adv.

[74] Shanker U, Rani M, Jassal V (2017). Environ Chem Lett.

[75] Carmen Z, Daniela S, Puzyn T, Mostrag-Szlichtyng A (2012). Textile Organic Dyes – Characteristics, Polluting Effects and Separation/Elimination Procedures from Industrial Effluents – A Critical Overview. Organic Pollutants Ten Years After the Stockholm Convention - Environmental and Analytical Update.

[76] Hosseini S A, Vossoughi M, Mahmoodi N M, Sadrzadeh M (2018). J Cleaner Prod.

[77] Hosseini S A, Vossoughi M, Mahmoodi N M, Sadrzadeh M (2019). Appl Clay Sci.

[78] Gopi S, Balakrishnan P, Pius A, Thomas S (2017). Carbohydr Polym.

[79] Huong D T M, Chai W S, Show P L, Lin Y-L, Chiu C-Y, Tsai S-L, Chang Y-K (2020). Int J Biol Macromol.

[80] Hou C, Jiao T, Xing R, Chen Y, Zhou J, Zhang L (2017). J Taiwan Inst Chem Eng.

[81] Liu Y, Hou C, Jiao T, Song J, Zhang X, Xing R, Zhou J, Zhang L, Peng Q (2018). Nanomaterials.

[82] Zeng G, He Y, Zhan Y, Zhang L, Pan Y, Zhang C, Yu Z (2016). J Hazard Mater.

[83] Yin H, Zhao J, Li Y, Huang L, Zhang H, Chen L (2020). J Cleaner Prod.

[84] Sundaran S P, Reshmi C R, Sagitha P, Manaf O, Sujith A (2019). J Environ Manage.

[85] Ma F-f, Zhang D, Huang T, Zhang N, Wang Y (2019). Chem Eng J.

[86] Li L, Wang F, Lv Y, Liu J, Zhang D, Shao Z (2018). Appl Clay Sci.

[87] Min L-L, Yang L-M, Wu R-X, Zhong L-B, Yuan Z-H, Zheng Y-M (2019). J Colloid Interface Sci.

[88] Shariful M I, Sepehr T, Mehrali M, Ang B C, Amalina M A (2018). J Appl Polym Sci.

[89] Homaeigohar S, Zillohu A U, Abdelaziz R, Hedayati M K, Elbahri M (2016). Materials.

[90] Habiba U, Siddique T A, Li Lee J J, Joo T C, Ang B C, Afifi A M (2018). Carbohydr Polym.

[91] Habiba U, Afifi A M, Salleh A, Ang B C (2017). J Hazard Mater.

[92] Zhang W, Yang P, Li X, Zhu Z, Chen M, Zhou X (2019). Int J Biol Macromol.

[93] Gao Q, Luo J, Wang X, Gao C, Ge M (2015). Nanoscale Res Lett.

[94] Stanly S, Jelmy E J, John H (2020). J Polym Environ.

[95] Chen H, Lin J, Zhang N, Chen L, Zhong S, Wang Y, Zhang W, Ling Q (2018). J Hazard Mater.

[96] Li Y, Zhao R, Chao S, Sun B, Wang C, Li X (2018). Chem Eng J.

[97] Tu H, Huang M, Yi Y, Li Z, Zhan Y, Chen J, Wu Y, Shi X, Deng H, Du Y (2017). Appl Surf Sci.

[98] Anis S F, Hashaikeh R, Hilal N (2019). J Water Process Eng.

[99] Gao W, Liang H, Ma J, Han M, Chen Z-l, Han Z-s, Li G-b (2011). Desalination.

[100] Anand A, Unnikrishnan B, Mao J-Y, Lin H-J, Huang C-C (2018). Desalination.

[101] Moradi G, Rajabi L, Dabirian F, Zinadini S (2018). J Appl Polym Sci.

[102] Moradi G, Zinadini S, Dabirian F, Rajabi L (2019). Sep Purif Technol.

[103] Zhang J, Xue Q, Pan X, Jin Y, Lu W, Ding D, Guo Q (2017). Chem Eng J.

[104] Islam M S, McCutcheon J R, Rahaman M S (2017). J Membr Sci.

[105] Jang W, Yun J, Jeon K, Byun H (2015). RSC Adv.

[106] Goetz L A, Jalvo B, Rosal R, Mathew A P (2016). J Membr Sci.

[107] Pishnamazi M, Koushkbaghi S, Hosseini S S, Darabi M, Yousefi A, Irani M (2020). J Mol Liq.

[108] Al-Husaini I S, Yusoff A R M, Lau W J, Ismail A F, Al-Abri M Z, Al-Ghafri B N, Wirzal M D H (2019). Sep Purif Technol.

[109] Zhijiang C, Cong Z, Ping X, Jie G, Kongyin Z (2018). J Mater Sci.

[110] Karunanidhi A, David P S, Fathima N N (2020). Water, Air, Soil Pollut.

[111] Malaeb L, Ayoub G M (2011). Desalination.

[112] Ghosh A K, Hoek E M V (2009). J Membr Sci.

[113] Wang X, Ma H, Chu B, Hsiao B S (2017). Desalination.

[114] Yang Y, Xu Y, Liu Z, Huang H, Fan X, Wang Y, Song Y, Song C (2020). J Membr Sci.

[115] Pan S-F, Ke X-X, Wang T-Y, Liu Q, Zhong L-B, Zheng Y-M (2019). Ind Eng Chem Res.

[116] Tian M, Wang Y-N, Wang R, Fane A G (2017). Desalination.

[117] Zhang C, Huang M, Meng L, Li B, Cai T (2017). J Chem Technol Biotechnol.

[118] Obaid M, Ghouri Z K, Fadali O A, Khalil K A, Almajid A A, Barakat N A M (2016). ACS Appl Mater Interfaces.

[119] Obaid M, Kang Y, Wang S, Yoon M-H, Kim C-M, Song J-h, Kim I S (2018). J Mater Chem A.

[120] Woo Y C, Kim Y, Shim W-G, Tijing L D, Yao M, Nghiem L D, Choi J-S, Kim S-H, Shon H K (2016). J Membr Sci.

[121] Koo J, Nam S-H, Kim E, Hwang T-M, Lee S (2017). Desalin Water Treat.

[122] Mericq J-P, Laborie S, Cabassud C (2011). Chem Eng J.

[123] Sarbatly R, Chiam C-K (2013). Appl Energy.

[124] Hou D, Lin D, Ding C, Wang D, Wang J (2017). Sep Purif Technol.

[125] Hou D, Ding C, Fu C, Wang D, Zhao C, Wang J (2019). Desalination.

[126] Dong Z-Q, Wang B-J, Ma X-h, Wei Y-M, Xu Z-L (2015). ACS Appl Mater Interfaces.

[127] Huang Y-X, Wang Z, Hou D, Lin S (2017). J Membr Sci.

[128] Lee E-J, An A K, He T, Woo Y C, Shon H K (2016). J Membr Sci.

[129] Liao Y, Wang R, Fane A G (2014). Environ Sci Technol.

[130] Prince J A, Singh G, Rana D, Matsuura T, Anbharasi V, Shanmugasundaram T S (2012). J Membr Sci.

[131] Yang F, Efome J E, Rana D, Matsuura T, Lan C (2018). ACS Appl Mater Interfaces.

[132] Li H, Shi W, Zeng X, Huang S, Zhang H, Qin X (2020). Sep Purif Technol.

[133] Elmarghany M R, El-Shazly A H, Rajabzadeh S, Salem M S, Shouman M A, Nabil Sabry M, Matsuyama H, Nady N (2020). Membranes.

[134] Fiorenza R, Bellardita M, D’Urso L, Compagnini G, Palmisano L, Scirè S (2016). Catalysts.

[135] Adams L K, Lyon D Y, Alvarez P J J (2006). Water Res.

[136] Chang X, Zhang Y, Tang M, Wang B (2013). Nanoscale Res Lett.

[137] Ognibene G, Cristaldi D A, Fiorenza R, Blanco I, Cicala G, Scirè S, Fragalà M E (2016). RSC Adv.

[138] Zhang X, Wang D K, Diniz da Costa J C (2014). Catal Today.

[139] Leong S, Razmjou A, Wang K, Hapgood K, Zhang X, Wang H (2014). J Membr Sci.

[140] Ademola Bode-Aluko C, Pereao O, Kyaw H H, Al-Naamani L, Al-Abri M Z, Tay Zar Myint M, Rossouw A, Fatoba O, Petrik L, Dobretsov S (2021). Mater Sci Eng, B.

[141] Blanco M, Monteserín C, Angulo A, Pérez-Márquez A, Maudes J, Murillo N, Aranzabe E, Ruiz-Rubio L, Vilas J L (2019). Polymers (Basel, Switz).

[142] Wu X-Q, Shao Z-D, Liu Q, Xie Z, Zhao F, Zheng Y-M (2019). J Colloid Interface Sci.

[143] Wu X-Q, Shen J-S, Zhao F, Shao Z-D, Zhong L-B, Zheng Y-M (2018). J Mater Sci.

[144] Salazar H, Martins P M, Santos B, Fernandes M M, Reizabal A, Sebastián V, Botelho G, Tavares C J, Vilas-Vilela J L, Lanceros-Mendez S (2020). Chemosphere.

[145] Yar A, Haspulat B, Üstün T, Eskizeybek V, Avcı A, Kamış H, Achour S (2017). RSC Adv.

[146] Li Q-H, Dong M, Li R, Cui Y-Q, Xie G-X, Wang X-X, Long Y-Z (2021). Carbohydr Polym.

[147] Campagnolo L, Lauciello S, Athanassiou A, Fragouli D (2019). Water.

[148] Rosman N, Wan Salleh W N, Aziz F, Ismail A F, Harun Z, Bahri S S, Nagai K (2019). Catalysts.

[149] Kanjwal M A, Chronakis I S, Barakat N A M (2015). Ceram Int.

[150] Chen H, Jiang G, Yu W, Liu D, Liu Y, Li L, Huang Q, Tong Z, Chen W (2016). Powder Technol.

[151] Zhong Y, Qiu X, Chen D, Li N, Xu Q, Li H, He J, Lu J (2016). ACS Appl Mater Interfaces.

[152] Liu H, Tang X, Liu Q (2014). J Water Health.

[153] Fahimirad S, Fahimirad Z, Sillanpää M (2021). Sci Total Environ.

[154] Parekh S A, David R N, Bannuru K K, Krishnaswamy L, Baji A (2018). Membranes.

[155] Shalaby T, Hamad H, Ibrahim E, Mahmoud O, Al-Oufy A (2018). Ecotoxicol Environ Saf.

[156] He J, Wang W, Shi R, Zhang W, Yang X, Shi W, Cui F (2018). Chem Eng J.

[157] Yang S, Lei P, Shan Y, Zhang D (2018). Appl Surf Sci.

[158] Wang J, Wu Y, Yang Z, Guo H, Cao B, Tang C Y (2017). Sci Rep.

[159] Xie L, Shu Y, Hu Y, Cheng J, Chen Y (2020). Chemosphere.

